# Jasmonate activates a CsMPK6-CsMYC2 module that regulates the expression of β-citraurin biosynthetic genes and fruit coloration in orange (*Citrus sinensis*)

**DOI:** 10.1093/plcell/koac363

**Published:** 2022-12-19

**Authors:** Pengtao Yue, Zhenghua Jiang, Quan Sun, Ranran Wei, Yingzi Yin, Zongzhou Xie, Robert M Larkin, Junli Ye, Lijun Chai, Xiuxin Deng

**Affiliations:** Key Laboratory of Horticultural Plant Biology of MOE (Ministry of Education), Huazhong Agricultural University Wuhan, Hubei 430070, China; Key Laboratory of Horticultural Plant Biology of MOE (Ministry of Education), Huazhong Agricultural University Wuhan, Hubei 430070, China; Key Laboratory of Horticultural Plant Biology of MOE (Ministry of Education), Huazhong Agricultural University Wuhan, Hubei 430070, China; Key Laboratory of Horticultural Plant Biology of MOE (Ministry of Education), Huazhong Agricultural University Wuhan, Hubei 430070, China; Key Laboratory of Horticultural Plant Biology of MOE (Ministry of Education), Huazhong Agricultural University Wuhan, Hubei 430070, China; Key Laboratory of Horticultural Plant Biology of MOE (Ministry of Education), Huazhong Agricultural University Wuhan, Hubei 430070, China; Key Laboratory of Horticultural Plant Biology of MOE (Ministry of Education), Huazhong Agricultural University Wuhan, Hubei 430070, China; Key Laboratory of Horticultural Plant Biology of MOE (Ministry of Education), Huazhong Agricultural University Wuhan, Hubei 430070, China; Key Laboratory of Horticultural Plant Biology of MOE (Ministry of Education), Huazhong Agricultural University Wuhan, Hubei 430070, China; Key Laboratory of Horticultural Plant Biology of MOE (Ministry of Education), Huazhong Agricultural University Wuhan, Hubei 430070, China; Hubei Hongshan Laboratory Wuhan, Hubei 430070, China

## Abstract

Carotenoids are natural pigments that influence the color of citrus fruit. The red-colored carotenoid β-citraurin is responsible for the peel color in “Newhall” orange (*Citrus sinensis*). Although jasmonates are known to regulate the biosynthesis and accumulation of carotenoids, their effects on β-citraurin biosynthesis in citrus fruit remain unclear. Here, we determined that treatment with methyl jasmonate (MeJA) significantly promotes fruit coloration and β-citraurin production in “Newhall” orange. A MeJA treatment induced the expression of *CsMYC2*, which encodes a transcription factor that serves as a master regulator of jasmonate responses. CsMYC2 bound the promoter of the gene that encodes carotenoid cleavage dioxygenase 4b (CsCCD4b), the key gene for β-citraurin biosynthesis, and the promoters of genes that encode phytoene synthase (CsPSY), lycopene β-cyclase (CsLCYb), and β-carotene hydroxylase (CsBCH) and induced their expression. In addition, CsMYC2 promoted *CsMPK6* expression. Notably, we found that CsMPK6 interacted with CsMYC2 and that this interaction decreased the stability and DNA-binding activity of CsMYC2. Thus, we conclude that negative feedback regulation attenuates JA signaling during the jasmonate-induced coloration of citrus fruit. Together, our findings indicate that jasmonates induce β-citraurin biosynthesis in citrus by activating a CsMPK6–CsMYC2 cascade, thereby affecting fruit coloration.

## Teaser

Jasmonate promotes the accumulation of the carotenoid β-citraurin in the peel of ‘Newhall’ orange fruit and thus, red-orange fruit coloration by activating the CsMPK6-CsMYC2 module and JA responses.

**IN A NUTSHELL**

**Background:** Beautiful, bright peel colors attract animals to help disperse seeds and are preferred by consumers. In agricultural production, citrus fruit often suffers from uneven coloration and poor development of color in the fruit peel, which limits fruit value. Carotenoids are natural pigments in plants and the red carotenoid β-citraurin is essential for the peel coloration of “Newhall” orange fruit. Although many signaling pathways are linked to the regulation of carotenoid biosynthesis, how β-citraurin biosynthesis is regulated during citrus fruit development and maturation is still poorly understood, which hampers the development of management strategies for improving citrus fruit coloration. Jasmonates regulate carotenoid biosynthesis, but their role in citrus fruit coloration is unknown.

**Question:** What are the detailed mechanisms by which jasmonates regulate β-citraurin biosynthesis?

**Findings:** We found that exogenous methyl jasmonate (MeJA) treatment of “Newhall” orange fruit promoted β-citraurin accumulation and fruit peel coloration. The jasmonate signaling master transcription factor CsMYC2 bound promoters of β-citraurin biosynthetic genes and activated their expression and promoted expression of the MAP kinase gene *CsMPK6*. In turn, CsMPK6 interacted with CsMYC2 to decrease the promoter-binding activity of CsMYC2 to its target promoters. CsMPK6 also phosphorylated CsMYC2 to accelerate its degradation and thus, attenuated the jasmonate response for citrus to prevent the fruit from overreacting to jasmonates.

**Next steps:** An important task for next step is to explore whether other signaling pathways interact with jasmonates to coregulate β-citraurin biosynthesis. With that work, we will investigate whether CsMYC2 serves as a mediator to bridge jasmonate signaling with other signaling pathways in regulating citrus coloration.

## Introduction

Carotenoids are widespread natural pigments with colors that mostly range from yellow to red that affect the coloration of fruits and vegetables, such as tomato (*Solanum lycopersicum*), carrot (*Daucus carota*), and citrus (*Citrus* spp.) (Nisar et al., 2015; Yuan et al., 2015). The development of peel color is a particularly important step in fruit development and maturation because brightly colored peels attract animals that help to disperse seeds, increase fruit value, and are preferred by consumers (Ruiz-Sola and Rodriguez-Concepcion, 2012; Yuan et al., 2015). The peel color in citrus including tangerine (*Citrus reticulata*), pomelo (*Citrus maxima*), and orange (*Citrus sinensis*) is determined by the levels and types of carotenoids present (Yuan et al., 2015). “Newhall” navel orange peels are enriched for the special, red-colored carotenoid β-citraurin, which is essential for the bright orange-red peel color (Yuan et al., 2015; Zheng et al., 2019).

β-citraurin accumulates in certain citrus cultivars and is a product of the carotenoid biosynthetic pathway (Ma et al., 2013; Rodrigo et al., 2013; Zheng et al., 2019). Briefly, the precursor geranylgeranyl diphosphate is used to synthesize lycopene in a process catalyzed by phytoene synthase (PSY) and a series of other enzymes (Nisar et al., 2015). Lycopene is in turn transformed to β-citraurin by lycopene β-cyclase (LCYb), β-carotene hydroxylase (BCH), and carotenoid cleavage dioxygenase (CCD) by means of a stepwise process (Liu et al., 2015; Zheng et al., 2019). The genes encoding these structural enzymes collectively influence fruit color. For example, silencing *SlPSY1* reduces the accumulation of all types of carotenoids, resulting in the production of fruit with yellow flesh in tomato (Fantini et al., 2013). In contrast, silencing *SlLCYB* leads to increases in lycopene levels and fruit with a deep red color (Ronen et al., 2000).

CCD is the last enzyme in the β-citraurin biosynthetic pathway. *CCD4b* expression was recently linked to β-citraurin accumulation in many citrus varieties (Ma et al., 2013; Zheng et al., 2019). In β-citraurin-enriched citrus, an A to G single nucleotide polymorphism (SNP) converts CAACT**A** to CAACT**G** in a miniature inverted repeat transposable element (MITE) that is inserted into the *CCD4b* promoter and thus, creates an E-box that enhances *CCD4b* expression. However, in varieties that do not accumulate β-citraurin and that express low levels of *CCD4b*, the *CCD4b* promoter either contains only the MITE without the SNP (CAACTA) or does not contain the MITE (Zheng et al., 2019). These findings provide evidence that low levels of *CCD4b* expression limit the rate of β-citraurin biosynthesis in citrus.

Transcription factors (TFs) and hormones regulate the expression of these structural genes. For example, in tomato, the MADS type TF RIPENING INHIBITOR (RIN) binds the *PSY* promoter and enhances its expression. The *rin* mutant produces yellow fruit that accumulates low levels of carotenoids (Li et al., 2017a; Li et al., 2020). In citrus, the TFs CsERF061, CsMADS6, and CrMYB68 contribute to the accumulation of carotenoids by regulating the expression of carotenoid biosynthetic genes (Zhu et al., 2017; Lu et al., 2018; Zhu et al., 2021a). Moreover, ethylene promotes fruit coloration in tomato and citrus by activating the expression of carotenoid biosynthetic genes (Marty et al., 2005; Fujisawa et al., 2012; Fujisawa et al., 2013; Luan et al., 2020; Sun et al., 2021).

Jasmonates regulate many biological processes in plants, including root elongation, stress responses, and carotenoid biosynthesis (Shan et al., 2009; Wasternack et al., 2013; An et al., 2021; Li et al., 2021). In tomato fruit, lycopene levels were reduced in jasmonate-deficient plants. In contrast, carotenoid accumulation and fruit coloration accelerated in fruits treated with exogenous methyl jasmonate (MeJA) (Tzortzakis and Economakis, 2010; Liu et al., 2012). Consistent with these data, a MeJA treatment promotes carotenoid biosynthetic gene expression in maize (*Zea mays*) (Luo et al., 2020).

The basic helix-loop-helix (bHLH) class of MYC-type TFs are central regulators of JA-related responses in plants (Li et al., 2021). MYC is activated by the recognition of JA and the JA receptor (Song et al., 2014; Wasternack and Song, 2017; Zander et al., 2020; Li et al., 2021). Jasmonate responses stimulate negative feedback regulators of jasmonate signaling, such as PUB (plant U-box protein), MPK (mitogen-activated protein kinase), and BPM (BTB/POZ-MATH) that interact with and attenuate the activity of MYC to prevent the overactivation of jasmonate responses (Zhai et al., 2013; Sethi et al., 2014; Jung et al., 2015; Wasternack, 2019; Chico et al., 2020; Li et al., 2021). MYC and inhibitors of jasmonate signaling collectively regulate the response to JA.

Jasmonates drive the accumulation of carotenoids in fruits by inducing the expression of carotenoid biosynthetic genes (Tzortzakis and Economakis, 2010; Liu et al., 2012; Luo et al., 2020). However, it is not clear whether the MYC TF associated with jasmonate signaling contributes to carotenoid biosynthesis and the development of fruit color in citrus by regulating β-citraurin biosynthesis. Here, we demonstrate that a treatment with exogenous MeJA significantly induced the expression of β-citraurin biosynthetic genes and fruit coloration in “Newhall” orange fruit by activating *CsMYC2* expression. We conclude that CsMYC2 activates *CsMPK6* expression, and in turn, that CsMPK6 binds CsMYC2, reduces the stability of CsMYC2, and attenuates the binding affinity of CsMYC2 for the promoters of genes involved in β-citraurin biosynthesis. These findings shed light on the mechanism used by JA to regulate carotenoid biosynthesis and coloration in citrus fruit.

## Results

### MeJA treatments promote β-citraurin accumulation and citrus fruit coloration

To explore the potential relationship between JA and the accumulation of β-citraurin in orange (cv. Newhall), we quantified the levels of endogenous jasmonic acid (JA), its bioactive form jasmonoyl-isoleucine (JA-Ile), its biosynthetic precursor 12-oxo-phytodienoic acid (OPDA), and β-citraurin in orange peels from the early stages of fruit development until the later stages of fruit maturation. We found that the JA and JA-Ile levels peaked at 210 DAFB (days after full blossom) and declined slowly until 240 DAFB (Supplemental Figure 1, A and B). In contrast, we observed that OPDA levels decreased gradually from 120 DAFB to 210 DAFB and increased at 240 DAFB—the opposite trend relative to JA and JA-Ile (Supplemental Figure 1C). The β-citraurin content only increased from 120 to 240 DAFB (Supplemental Figure 1D). We examined the levels of *CsCCD4b* mRNA during fruit development and maturation using reverse-transcription-quantitative PCR (RT-qPCR). The expression pattern of this gene resembled the pattern of β-citraurin accumulation (Supplemental Figure 1E). These results provide evidence that JA promotes the accumulation of β-citraurin.

**Figure 1:**
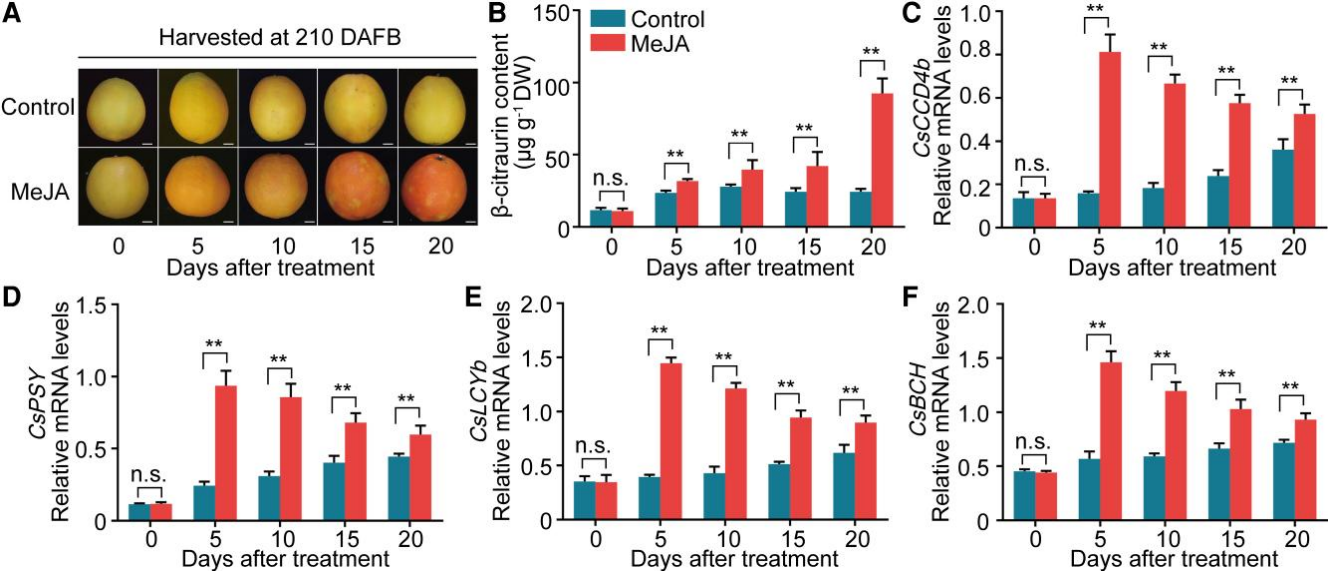
Jasmonate promotes β-citraurin biosynthesis and citrus fruit coloration. A–F, “Newhall” orange fruit was harvested at 210 DAFB (days after full blossom) and treated with MeJA, stored at room temperature for 20 d, and fruit peels were sampled every 5 d (A, phenotype of treated fruit). Untreated fruit was used as a control. Scale bars, 1 cm. β-citraurin concentration was measured (B) and the mRNA levels of *CsCCD4b* (C), *CsPSY* (D), *CsLCYb* (E), and *CsBCH* (F) were determined using RT-qPCR. Numbers under the *X* axes in (B) to (F) indicate the DAT. At the sampling time, peel samples from each treatment were divided into three sets. Each set contained peels from three fruits. An independent RNA or carotenoid extraction from each set of peels was used as one biological replicate. Three replicates were performed. Values represent means ± SE. Asterisks indicate statistically significant differences, as determined by a Student's *t* test (**P < .01). n.s., no significant difference.

We sprayed fruit on the tree with different concentrations (0.2, 0.5, 1, and 4 mM) of MeJA at 180 DAFB. We observed that MeJA accelerated color development in citrus fruit when it was applied at a concentration of 0.5 mM and that the fruit peel was damaged considerably when the concentration was increased to 4 mM (Supplemental Figure 2). In addition, we harvested fruit at 210 DAFB, treated it with 0.5 mM MeJA, and stored it at room temperature for 20 d with sampling every 5 d (Figure 1A). During storage, MeJA treatment significantly promoted the accumulation of β-citraurin and fruit coloration, especially after 15 days after treatment (DAT) (Figure 1, A and B). The accumulation of mRNA from *jasmonic acid resistant 1* (*CsJAR1*), a JA-responsive gene, and the endogenous levels of both JA and JA-Ile were upregulated. These data indicate that the MeJA treatment of orange fruit was successful (Supplemental Figure 3, A–C). We examined the levels of mRNA expressed from β-citraurin biosynthetic genes. The expression of *CsCCD4b*, *CsPSY*, *CsLCYb*, and *CsBCH* were upregulated in MeJA-treated fruit relative to the control (Figure 1, C–F). Their expression levels were highest at 5 DAT and declined thereafter (Figure 1, C–F). Finally, we measured the levels of carotenoids that contribute to β-citraurin biosynthesis (Supplemental Figure 3D). MeJA treatment strongly induced the production of β-carotene. In contrast, β-carotene accumulated to barely detectable levels in the control fruit (Supplemental Figure 3E). β-cryptoxanthin and violaxanthin were both highly enriched in MeJA-treated fruit compared to control fruit (Supplemental Figure 3, F and G). These results indicate that JA promotes fruit coloration in “Newhall” orange by enhancing β-citraurin biosynthesis.

Ethylene and abscisic acid (ABA) are important plant hormones that influence carotenoid biosynthesis (Liu et al., 2015; Yuan et al., 2015; Enfissi et al., 2017; Sun et al., 2021). To determine whether JA regulates β-citraurin biosynthesis and fruit coloration by influencing the ethylene or ABA pathways, we treated Lane Late Navel orange and Orah mandarin fruits, two citrus varieties that accumulate β-citraurin (Zhu et al., 2021a; Huang et al., 2022), with either the ethylene inhibitor 1-methylcyclopropene (1-MCP), or the ABA inhibitor nordihydroguaiaretic acid (NDGA) to disrupt ethylene signaling and ABA biosynthesis. The 1-MCP-treated fruit and the NDGA-treated fruit were then treated with MeJA (1-MCP/MeJA, NDGA/MeJA). In both varieties, the peel colors of the 1-MCP-treated and NDGA-treated fruits were similar. However, the MeJA treatment promoted the coloration of fruits regardless of whether the fruits were pretreated with either 1-MCP or NDGA (Supplemental Figure 4A).

To obtain mechanistic insight into this phenomenon, we quantified the levels of the ethylene precursor ACC and ABA in MeJA-treated and untreated “Newhall” orange fruit that was harvested at 210 DAFB. The MeJA treatment did not influence the accumulation of ACC but increased ABA levels in “Newhall” orange fruit (Supplemental Figure 4, B and C). Next, we quantified the levels of JA, JA-Ile, ACC, and ABA in MeJA-treated and untreated Lane Late Navel orange and Orah mandarin fruits. In both varieties, the MeJA treatment increased JA and JA-Ile levels regardless of whether the fruits had been pretreated with either 1-MCP or NDGA (Supplemental Figure 4, D, E, H, and I). ACC levels decreased in the 1-MCP-treated fruits relative to the control fruits. The application of MeJA had little impact on the accumulation of ACC (Supplemental Figure 4, F and J). ABA levels were suppressed by the NDGA treatments. Treatments with exogenous MeJA failed to promote the accumulation of ABA (Supplemental Figure 4, G and K). Collectively, these data provide evidence that jasmonates can directly regulate β-citraurin biosynthesis and fruit coloration by activating a mechanism that functions independently of ethylene and ABA.

### MeJA treatment promotes *CsMYC2* expression and upregulates the expression of β-citraurin biosynthetic genes

To investigate the mechanism used by jasmonates to regulate β-citraurin biosynthesis in citrus, we searched the citrus genome for TFs that serve as master regulators of jasmonate signaling using PLAZA (https://bioinformatics.psb.ugent.be/plaza/), Phytozome (https://phytozome-next.jgi.doe.gov/), and CPBD (http://citrus.hzau.edu.cn/). We identified three *MYC* genes (*CsMYC1/2/3*) and quantified the levels of mRNA expressed from these genes in citrus peels at 180 DAFB (yellow color) and 240 DAFB (red color) using RT-qPCR to determine whether any of these *MYC* genes might participate in β-citraurin biosynthesis during fruit development and maturation. Only *CsMYC2* was highly expressed in the red peel samples at 240 DAFB. In contrast, the expression of *CsMYC1* and *CsMYC3* remained extremely low in both yellow and red peel samples (Supplemental Figure 5A).

**Figure 2:**
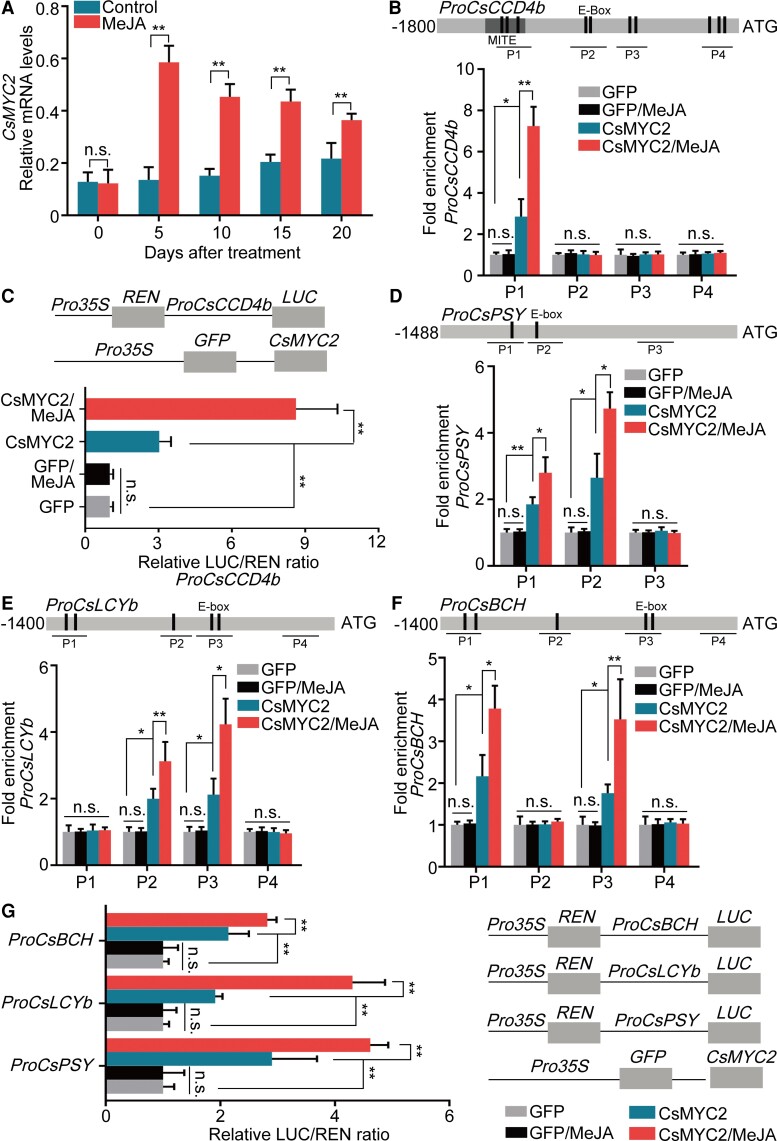
JA-activated *CsMYC2* positively regulates the expression of β-citraurin biosynthetic genes. A, *CsMYC2* mRNA levels in fruit peels. Relative mRNA accumulation was quantified using RT-qPCR. Fruit peel samples were the same as in Figure 1A. Control, untreated fruit. MeJA, MeJA-treated fruit. Numbers under the *x* axis indicate the number of DAT. Peel samples from each treatment were divided into three sets. Each set contained peels from three fruits. An independent RNA extraction from each set of peels was used as one biological replicate. Three replicates were performed. Values represent means ± SE. Asterisks indicate statistically significant differences, as determined by a Student's *t*- test (**P < .01). n.s., no significant difference. B, *CsCCD4b* promoter-binding activity of CsMYC2 in vivo. ChIP-PCR analysis was performed with an 1800-bp fragment from the *CsCCD4b* promoter. Chromatin was cross-linked and isolated from CsMYC2-GFP (CsMYC2)-overexpressing “Newhall” orange calli that was either untreated or treated with MeJA. The ChIP was performed with a GFP antibody. Sequences harboring E-box elements (i.e. bHLH binding sites) were amplified from the immunoprecipitated DNA using qPCR. Four fragments (P1–P4) were detected. Calli expressing GFP alone were used as negative controls. Three infected lines of callus were used for ChIP-PCR experiments. Each line was used as one biological replicate. The enriched DNA fragments from each line were analyzed using qPCR. Three biological replicates were performed. The enrichment from the GFP negative control was defined as 1. The final enrichment results are represented as the fold enrichment relative to the GFP negative control. The ChIP data are an average from three biological replicates. Values represent means ± SE. Asterisks indicate statistically significant differences, as determined by a Student's *t* test (*P < .05, **P < .01). n.s., no significant difference. C, Transactivation activity of CsMYC2 on the *CsCCD4b* promoter. CsMYC2 effector and *CsCCD4b* promoter-*LUC* reporter plasmids were coinfiltrated into *N. benthamiana* leaves to analyze LUC activity. Three independent transfection replicates were analyzed. Values represent means ± SE. Asterisks indicate statistically significant differences as determined by a Student's *t* test (**P < .01). n.s., no significant difference. D, CsMYC2 binding the *CsPSY* promoter (1,488 bp) in vivo. ChIP-PCR assays were performed as described in (B). Three fragments (P1–P3) were analyzed from three biological replicates using the ChIP assay. Values represent means ± SE. Asterisks indicate statistically significant differences, as determined by a Student's *t* test (*P < .05, **P < .01). n.s., no significant difference. E, CsMYC2 binding the *CsLCYb* promoter (1,400 bp) in vivo. ChIP-PCR assays were performed as described in (B). Four fragments (P1–P4) were analyzed from three biological replicates using the ChIP assay. Values represent means ± SE. Asterisks indicate statistically significant differences, as determined by a Student's *t* test (*P < .05, **P < .01). n.s., no significant difference. F, CsMYC2 binding the *CsBCH* promoter (1,400 bp) in vivo. ChIP-PCR assays were performed as described in Figure 2B. Four fragments (P1–P4) were analyzed from three biological replicates using the ChIP assay. Values represent means ± SE. Asterisks indicate statistically significant differences, as determined by a Student's *t* test (*P < .05, **P < .01). n.s., no significant difference. G, Transactivation activity of CsMYC2 on *CsPSY*, *CsLCYb*, and *CsBCH*. The CsMYC2 effector plasmid was separately coinfiltrated with plasmids containing the *CsPSY*, *CsLCYb*, and *CsBCH* promoter-driven *LUC* reporter genes into *N. benthamiana* leaves to quantify LUC activity. Three independent infiltration replicates were analyzed. Values represent means ± SE. Asterisks indicate statistically significant differences, as determined by a Student's *t* test (**P < .01). n.s., no significant difference.

The pattern of β-citraurin accumulation and the temporal expression patterns of *CsMYC2* and *CsCCD4b* were similar during fruit development and maturation (Supplemental Figure 1, D, E, and 5B). To test whether *CsMYC2* expression is regulated by jasmonates, we disrupted JA biosynthesis in “Newhall” orange fruits on the tree at 160 DAFB using the JA biosynthesis inhibitor sodium diethyldithiocarbamate (DIECA). We observed that the DIECA treatment reduced the levels of endogenous OPDA, JA, and JA-Ile, reduced the expression of *CsMYC2* and reduced the levels of β-citraurin (Supplemental Figure 5, C–E). We then examined the expression of *CsMYC2* in MeJA-treated fruit that was harvested at 210 DAFB. MeJA induced increases in the levels of *CsMYC2* mRNA. *CsMYC2* mRNA reached peak levels at 5 DAT followed by a slow decline (Figure 2A). We therefore chose *CsMYC2* as the candidate gene for further analysis. To examine the subcellular localization of CsMYC2, we used the *35S* promoter to transiently express a *CsMYC2-GFP* fusion gene in *Nicotiana benthamiana* leaves using *Agrobacterium*-mediated transformation. Confocal laser-scanning microscopy indicated that CsMYC2-GFP localized to the nucleus (Supplemental Figure 6).

We hypothesized that *CsMYC2* functions upstream of β-citraurin biosynthetic genes because the expression patterns of *CsMYC2*, *CsCCD4b*, *CsPSY*, *CsLCYb*, and *CsBCH* were similar during a MeJA treatment (Figures 1, C–F, and 2A). We previously reported that a SNP introduced into the *CCD4b* promoter by a MITE insertion, created an E-box (i.e. a bHLH binding site) that enhances *CCD4b* expression and β-citraurin accumulation in “Newhall” orange (Zheng et al., 2019). We inserted the coding sequence (CDS) of *CsMYC2* into pGADT7 (CsMYC2-Ad) and tested whether this TF binds the *CsCCD4b* promoter using a yeast one-hybrid (Y1H) assay, which confirmed that CsMYC2 binds the *CsCCD4b* promoter (Supplemental Figure 7A). We performed an electrophoretic mobility shift assay (EMSA) to test whether CsMYC2 binds the E-box in the *CsCCD4b* promoter that was created by the MITE insertion. We found that CsMYC2 bound the E-box element and that the mobility shift was impaired by the unlabeled competitor (Supplemental Figure 7B). Finally, we quantified the binding affinity of CsMYC2 for the *CsCCD4b* promoter using a biolayer interferometry (BLI) assay. The *K*_d_ value was 58 nM, which demonstrates that CsMYC2 can bind the *CsCCD4b* promoter (Supplemental Figure 7C). These data indicate that CsMYC2 binds this E-box in the *CsCCD4b* promoter in vitro.

To determine whether this binding occurs in vivo, we performed a chromatin immunoprecipitation (ChIP)-PCR assay. CsMYC2-GFP was transiently overexpressed in “Newhall” citrus calli (CsMYC2) using *Agrobacterium*-mediated transformation. Calli overexpressing GFP were used as a negative control (GFP). Using quantitative PCR (qPCR), we detected a strong signal from fragment P1, which includes the E-box introduced by the MITE. These data indicate that CsMYC2 binds the *CsCCD4b* promoter in vivo (Figure 2B). MeJA treatment enhanced this binding (Figure 2B). We explored the regulation of the *CsCCD4b* promoter by CsMYC2 using a Dual-LUC (luciferase) activity assay. When *CsMYC2-GFP* and *ProCsCCD4b:LUC* were coexpressed in *N. benthamiana* leaves and “Newhall” citrus calli, a significantly higher LUC ratio was obtained in the presence of CsMYC2-GFP, indicating that this TF positively regulates *CsCCD4b* promoter activity (Figure 2C; Supplemental Figure 8A). This activation was enhanced by a MeJA treatment (Figure 2C; Supplemental Figure 8A).

We examined the binding and regulation of the *CsPSY*, *CsLCYb*, and *CsBCH* promoters by CsMYC2. We analyzed the promoters of these genes using PLACE (https://www.dna.affrc.go.jp/PLACE/) and identified several E-boxes. We synthesized labeled probes based on the E-boxes closest to the translation start sites and used these probes to perform EMSAs. CsMYC2 bound the promoters of all three genes in EMSAs (Supplemental Figure 7, D–F) and in ChIP-PCR assays (Figures 2, D–F). LUC activity assays demonstrated that CsMYC2 positively regulates the expression of these β-citraurin biosynthetic genes, which was enhanced by a MeJA treatment (Figure 2G; Supplemental Figure 8, B–D). These results indicate that JA-activated CsMYC2 promotes the expression of β-citraurin biosynthetic genes by binding their promoters.

### CsMYC2 promotes *CsMPK6* expression and interacts with CsMPK6

After a MeJA treatment, the expression levels of the β-citraurin biosynthetic genes decreased and β-citraurin levels increased (Figure 1). Moreover, we found that the expression levels of *CsMYC2* and the JA-responsive gene *CsJAR1* paralleled the expression levels of β-citraurin biosynthetic genes (Figure 2A; Supplemental Figure 3A). These data indicate an attenuated response of citrus to JA. We hypothesized that a negative feedback mechanism might be activated during the jasmonate-induced coloration of citrus. PUBs, BPMs, and MPKs negatively regulate jasmonate signaling by interacting with MYC TFs in *Arabidopsis thaliana* (Zhai et al., 2013; Jung et al., 2015; Chico et al., 2020). We screened the citrus genome and identified 14 PUBs, 5 BPMs, 18 CsMPKs and analyzed their expression using the 5 DAT samples that were harvested at 210 DAFB and treated with MeJA (Supplemental Figure 9). We conducted Y2H (yeast two-hybrid) assays to test whether CsMYC2 interacts with proteins that accumulate to significantly elevated levels following MeJA treatment. We found that neither CsMYC2 nor CsMPK6 could serve as bait due to the strong autoactivation activities of their CDSs when they were expressed in yeast cells from the pGBKT7 vector. Therefore, we used CsMYC2-Ad as prey and expressed other proteins from pGBKT7 as bait, with the exception of CsMPK6, to test their interactions in yeast cells. No protein–protein interactions were observed (Supplemental Figure 10).

**Figure 3:**
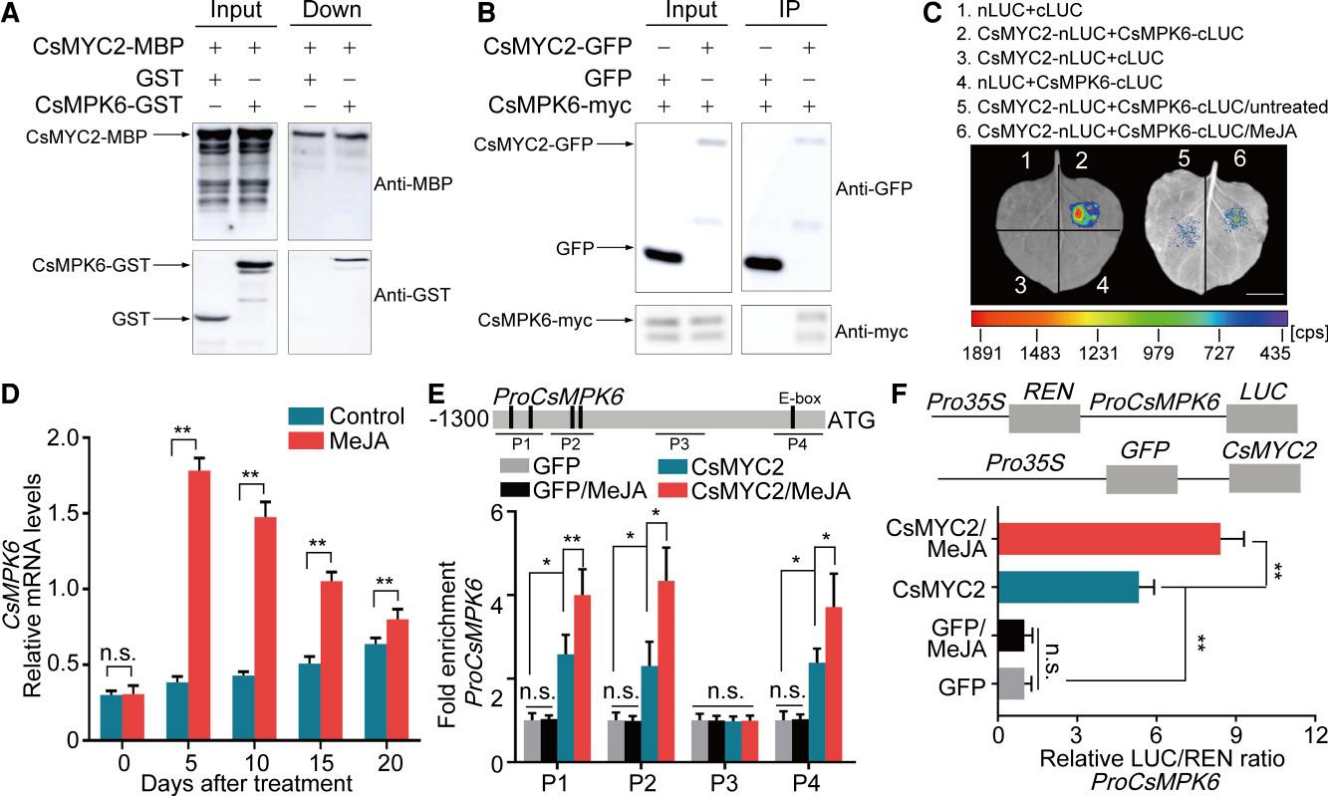
CsMYC2 binds CsMPK6 and transcriptionally activates *CsMPK6*. A, Interactions between CsMYC2 and CsMPK6 analyzed using a pull-down assay. The CsMYC2-MBP and CsMPK6-GST fusion proteins were expressed in *E. coli* and purified. CsMYC2-MBP was bound to MBP magnetic beads. The assays were analyzed using immunoblotting with anti-GST and anti-MBP antibodies. The band detected by the anti-GST antibody in the pulled down samples indicates an interaction between CsMYC2 and CsMPK6. B, Interactions between CsMYC2 and CsMPK6 analyzed using a CoIP assay. GFP-tagged CsMYC2 and myc-tagged CsMPK6 were co-overexpressed in *N. benthamiana* leaves. Anti-GFP magnetic beads were used for immunoprecipitation. Anti-GFP and anti-myc antibodies were used for immunoblotting analysis. Co-overexpression of GFP and CsMYC2-myc was used as a control. The band detected by the anti-Myc antibody in the IP samples indicates an interaction between CsMYC2 and CsMPK6. C, Interactions between CsMYC2 and CsMPK6 enhanced by a MeJA treatment. Different regions of *N. benthamiana* leaves were infiltrated with *Agrobacterium* strain EHA105 containing different constructs. Chemiluminescence from these regions produced by luciferase was imaged 3 d after infiltration. The MeJA treatment was performed 1 h before imaging. Scale bar, 1 cm. cps, signal counts per second. D, *CsMPK6* mRNA levels in fruit peels. Relative mRNA accumulation was quantified using RT-qPCR. Fruit peel samples were the same as in Figure 1A. Control, untreated fruit. MeJA, MeJA-treated fruit. The numbers under the *x* axis indicate the number of DAT. Peel samples from each treatment were divided into three sets. Each set contained peels from three fruits. An independent RNA extraction from each set of peels was used as one biological replicate. Three replicates were performed. Values represent means ± SE. Asterisks indicate statistically significant differences, as determined by a Student's *t* test (**P < .01). n.s., no significant difference. E, CsMYC2 binding the *CsMPK6* promoter (1,300 bp) in vivo. The ChIP-PCR assay was performed as in Figure 2B. Four fragments (P1–P4) were detected. Three biological replicates were analyzed using the ChIP assay. Values represent means ± SE. Asterisks indicate statistically significant differences, as determined by a Student's *t* test (*P < .05, **P < .01). n.s., no significant difference. F, CsMYC2 transactivates the *CsMPK6* promoter. To analyze LUC activity, *N. benthamiana* leaves were coinfiltrated with *Agrobacterium* strains harboring the CsMYC2 effector and the *CsMPK6* promoter-driven reporter gene. Three independent transfection replicates were analyzed. Values represent means ± SE. Asterisks indicate statistically significant differences, as determined by a Student's *t* test (**P < .01). n.s., no significant difference.

We separately expressed the CDSs from *CsMYC2* and *CsMPK6* as MBP and GST fusion proteins and tested whether these fusion proteins interact in pull-down assays. We found that CsMPK6-GST was pulled down by CsMYC2-MBP, which verified that they interact (Figure 3A). To validate this interaction, we performed a coimmunoprecipitation (CoIP) assay. We fused the CDS from *CsMPK6* to the CDS of a myc tag, coexpressed the CsMPK6-myc and CsMYC2-GFP fusion proteins in *N. benthamiana* leaves and found that CsMPK6 interacted with CsMYC2 in vivo (Figure 3B). Finally, we performed a LUC complementation experiment by fusing the CDSs from *CsMYC2* and *CsMPK6* with either the *N*- or C-terminus of LUC and coinfiltrated *N. benthamiana* leaves with these constructs. Strong chemiluminescent signals were detected, which confirms that CsMPK6 and CsMYC2 interact (Figure 3C, area 2). MeJA treatment enhanced this interaction (Figure 3C, area 5 and 6). To identify the CsMPK6-binding domain in CsMYC2, we separated CsMYC2 into the *N*-terminal domain containing the bHLH superfamily domain and the C-terminal domain containing the DNA-binding domain. A pull-down experiment showed that both the N- (lanes 1 and 2) and C-termini (lanes 3 and 4) of CsMYC2 bind CsMPK6 (Supplemental Figure 11, lanes 6 and 8). These results indicate that CsMYC2 interacts specifically with CsMPK6 and not with CsPUB, CsBPM, or other CsMPK proteins.

We measured *CsMPK6* mRNA levels in fruit that was harvested at 210 DAFB and treated with MeJA using RT-qPCR. The expression patterns of *CsMPK6* and *CsMYC2* were the same (Figure 3D). To determine whether *CsMPK6* expression might be regulated by CsMYC2, we analyzed its promoter using PLACE and identified five E-boxes. EMSAs and ChIP-PCR experiments showed that CsMYC2 bound the *CsMPK6* promoter in vitro and in vivo (Figure 3E; Supplemental Figure 7G) and that this binding was enhanced by a MeJA treatment (Figure 3E). A LUC activity assay indicated that transcription from the *CsMPK6* promoter was activated by CsMYC2. A MeJA treatment intensified this activation (Figure 3F; Supplemental Figure 8E). These data demonstrate that CsMYC2 binds and upregulates the expression of the JA signaling inhibitor gene *CsMPK6* and that CsMYC2 interacts with the CsMPK6 protein.

### CsMPK6 phosphorylates CsMYC2 and decreases its stability

**Figure 4:**
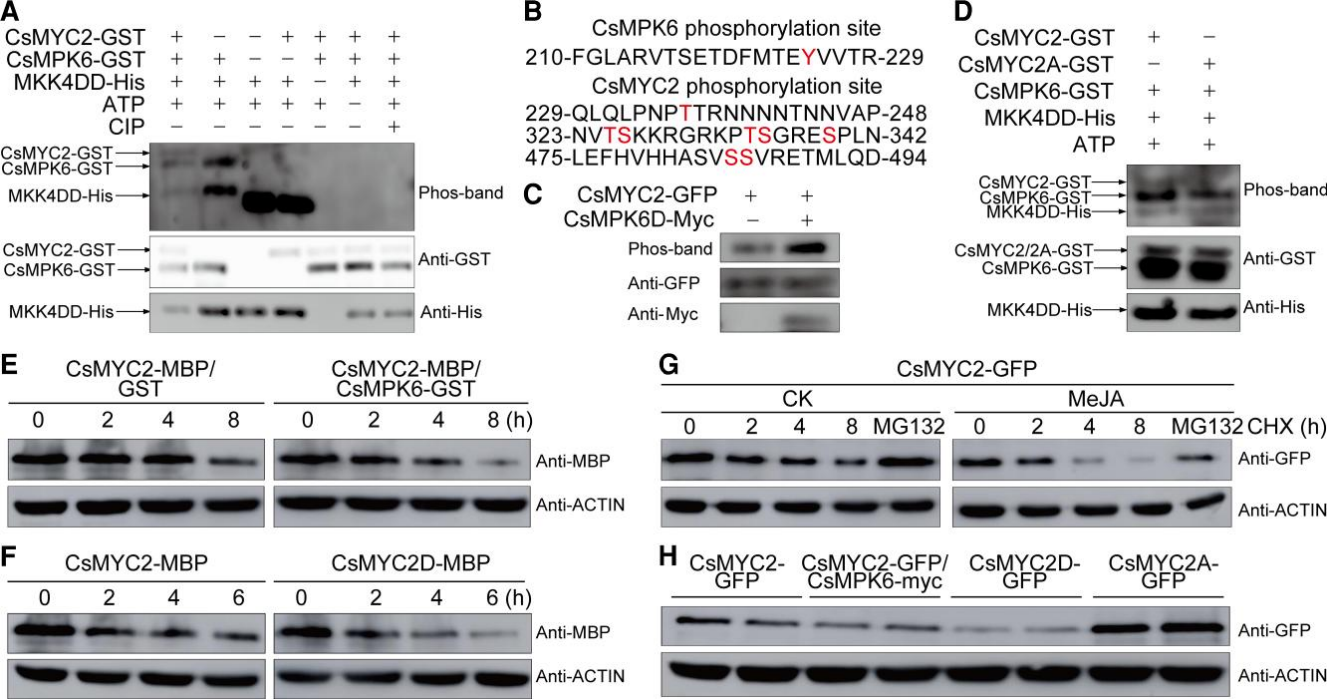
CsMPK6 phosphorylates CsMYC2 and decreases its stability. A, In vitro phosphorylation of CsMYC2 by CsMPK6. In vitro phosphorylation reactions were performed using the purified proteins as indicated. The top panel shows the CsMYC2 phosphorylation detected using Phos-bind Biotin BTL-104. The middle and bottom panels indicate the protein loading for the top panel detected using anti-GST and anti-His antibodies, respectively. B, Phosphorylation sites on CsMPK6 and CsMYC2. C, In vivo phosphorylation of CsMYC2 by CsMPK6. CsMYC2-GFP and CsMPK6D-myc (i.e. the phosphomimetic form of CsMPK6) were co-overexpressed in *N. benthamiana* leaves. Anti-GFP magnetic beads were used to immunoprecipitate the CsMYC2-GFP protein for Phos-bind biotin detection (top panel). Overexpression of CsMYC2-GFP alone was used as a control. Anti-GFP and anti-myc antibodies were used to indicate the protein loading of CsMYC2-GFP (middle panel) and CsMPK6-myc (bottom panel). D, CsMPK6 fails to phosphorylate CsMYC2A. *In vitro* phosphorylation reactions were performed using the purified proteins as indicated. The top panel shows phosphorylation analysis of CsMYC2 using Phos-bind Biotin BTL-104 for CsMYC2 and CsMYC2A, in which all phosphorylation sites were changed to A. The middle and bottom panels show immunoblots performed with anti-GST and anti-His antibodies that indicate the amount of protein loaded. E, Kinetic analysis of CsMYC2 degradation. Total proteins from 5 DAT samples that were harvested at 210 DAFB and treated with MeJA were extracted and mixed. Equal amounts of CsMYC2-MBP and equal amounts of total proteins were incubated with GST or CsMPK6-GST in the in vitro cell-free degradation assays for the times indicated. Anti-ACTIN was used as a loading control. F, Time-course of the degradation of CsMYC2-MBP and CsMYC2D-MBP (i.e. the phosphomimetic form of CsMYC2). The total protein mixtures were the same as in E, Equal amounts of recombinant proteins were incubated with equal amounts of total protein in the in vitro cell-free degradation system. Anti-ACTIN was used as a loading control. G, Time-course of the degradation of CsMYC2 treated with MeJA. “Newhall” orange calli that expressed MYC2-GFP were treated with MeJA followed by cycloheximide (CHX) and sampled at the times indicated. CsMYC2-GFP-overexpressing calli treated with DMSO were used as a control (CK). Calli additionally treated with MG132 were sampled at 8 h. The samples were analyzed by immunoblotting with anti-GFP antibodies. Actin (i.e. the loading control) was detected using anti-ACTIN antibodies. H, Protein levels of CsMYC2 in “Newhall” orange calli expressing CsMYC2-GFP, CsMYC2-GFP with CsMPK6-myc, CsMYC2D-GFP, and CsMYC2A-GFP. CsMYC2 was detected using immunoblotting with anti-GFP antibodies. The anti-ACTIN antibodies were used to quantify the total amount of protein loaded in each lane. Data from two independent pools of citrus calli are shown.

MPKs are important protein kinases. Since MKK4 is required for the phosphorylation activity of MPK6 (Shao et al., 2020), we inserted the CDS for MKK4DD (i.e. a phosphomimetic form of MKK4) into the pGBKT7 vector and performed a Y2H assay to test for interactions between MKK4DD and CsMPK6. MKK4DD interacted with CsMPK6, which is consistent with this kinase activating CsMPK6 (Supplemental Figure 12). We then investigated whether CsMPK6 phosphorylates CsMYC2. We expressed a GST-CsMYC2 fusion protein and a His-tagged MKK4DD in *E. coli* and purified them. We used the purified GST-CsMYC2, GST-CsMPK6, and His-MKK4DD fusion proteins in phos-band biotin assays. The phos-bind biotin probe detected the phosphorylated form of CsMYC2 when CsMYC2 protein was coincubated with both CsMPK6 and MKK4DD and was not detected if either CsMPK6 or MKK4DD was excluded from the assay. The signal from CsMYC2 was also eliminated when the sample was treated with calf intestinal alkaline phosphatase (Figure 4A). These data indicate that CsMPK6 phosphorylates CsMYC2.

We analyzed the phosphorylation sites on CsMPK6 and CsMYC2. We found that Y225 in CsMPK6 is phosphorylated and that T236, T325, S326, T334, S335, S339, S485, and S486 in CsMYC2 are phosphorylated (Figure 4B). We examined the phosphorylation between CsMPK6 and CsMYC2 in vivo by using site-directed mutagenesis to change Y225 to D225 (i.e. to produce a phosphomimetic form of CsMPK6 that we named CsMPK6D). We coexpressed a myc-tagged CsMPK6D and a CsMYC2-GFP fusion protein in *N. benthamiana* leaves. The phosphorylation of CsMYC2-GFP was significantly enhanced when CsMPK6D was coexpressed with CsMYC2-GFP (Figure 4C). Using site-directed mutagenesis, we changed the phosphorylation sites in CsMYC2 to A (i.e. produced a nonphosphorylatable form of CsMYC2 that we named CsMYC2A) and performed phos-band biotin assays. We did not observe a phospho-signal from CsMYC2A (Figure 4D). These data indicate that CsMYC2 is phosphorylated at these sites and that CsMYC2 is phosphorylated by CsMPK6 in vitro and in vivo.

To investigate the effect of phosphorylation on the stability of CsMYC2, we performed a cell-free protein degradation assay (He et al., 2020). CsMYC2 was degraded rapidly when CsMPK6 was added to the reaction (Figure 4E). When CsMYC2D (i.e. a phosphomimetic form of CsMYC2 with phosphorylation sites changed to D) was used in this system, CsMYC2 was degraded even more rapidly (Figure 4F). To study the degradation of CsMYC2 in vivo, we transiently overexpressed CsMYC2-GFP in “Newhall” citrus calli and treated the calli with MeJA and cycloheximide (CHX) to inhibit the synthesis of new proteins. The MeJA treatment significantly promoted the degradation of CsMYC2, and a MG132 treatment attenuated this degradation (Figure 4G). We also examined the accumulation of the CsMYC2 protein in calli that transiently overexpressed CsMYC2-GFP, CsMYC2D-GFP, CsMYC2A-GFP, or CsMYC2-GFP with CsMPK6-myc. CsMYC2A accumulated to the highest levels. The levels of CsMYC2 were significantly reduced by the co-overexpression of CsMPK6 relative to calli overexpressing only CsMYC2. The phosphomimetic form of CsMYC2, CsMYC2D, accumulated to the lowest levels (Figure 4H). These results indicate that phosphorylation decreases the stability of CsMYC2.

### CsMPK6 inhibits the promoter-binding activity of CsMYC2

**Figure 5:**
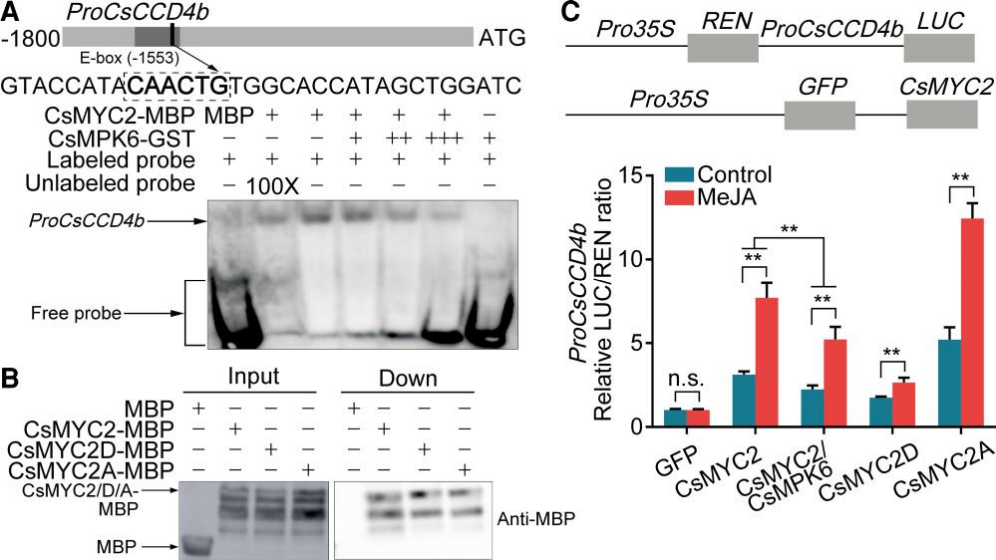
CsMPK6 inhibits the binding and transactivation activity of CsMYC2 on the *CsCCD4b* promoter. A, CsMPK6 attenuates the *CsCCD4b* promoter-binding activity of CsMYC2. EMSAs were conducted with a biotin-labeled *CsCCD4b* promoter fragment containing an E-box. An unlabeled version of the same *CsCCD4b* promoter fragment was used as a competitor at a 100-fold greater concentration than the labeled probe. MBP-tagged CsMYC2 protein (2 μg) and GST-tagged CsMPK6 protein (2, 6, 10 μg) were purified. Purified MBP (2 μg) was used as a negative control. B, No influence of phosphorylation on the DNA-binding activity of CsMYC2. Biotin-labeled *CsCCD4b* promoter probes were used to pull down the CsMYC2-MBP, CsMYC2D-MBP (i.e. the phosphomimetic form of CsMYC2), and CsMYC2A-MBP (i.e. the nonphosphorylatable form of CsMYC2) fusion proteins. Immunoblotting utilized the MBP-antibody. MBP was used as a control. C, CsMPK6 binds and inhibits the transactivation activity of CsMYC2 on the *CsCCD4b* promoter. Combinations of CsMYC2-GFP, CsMPK6-myc, CsMYC2D-GFP, and CsMYC2A-GFP were coinfiltrated with the *CsCCD4b* promoter-driven *LUC* reporter gene into *N. benthamiana* leaves before analyzing LUC activity. Three independent infiltration replicates were analyzed. Values represent means ± SE. Asterisks indicate statistically significant differences, as determined by a Student's *t* test (**P < .01). n.s., no significant difference.

We investigated the effects of CsMPK6 on CsMYC2 protein function by performing a DNA pull-down assay. When we incubated a biotin-labeled *CsCCD4b* promoter fragment (i.e. the same fragment used as a labeled probe in the EMSAs) with only the CsMYC2-MBP fusion protein or with both CsMYC2-MBP and CsMPK6-GST, probe–protein complexes were pulled down with streptavidin-conjugated magnetic beads. CsMPK6 significantly reduced CsMYC2 protein abundance and thus, suppressed the binding of CsMYC2 to the *CsCCD4b* promoter (Supplemental Figure 13A). Moreover, the addition of CsMPK6 to an EMSA reduced the binding of CsMYC2 to the *CsCCD4b* promoter fragment (Figure 5A). We also compared the DNA-binding activity of different forms of the CsMYC2 protein using DNA pull-down assays and EMSAs. We found no difference among CsMYC2, CsMYC2D, and CsMYC2A (Figure 5B; Supplemental Figure 13B). These results indicated that CsMPK6 inhibits the binding of CsMYC2 to its target promoter by protein–protein interactions rather than by phosphorylating CsMYC2.

In addition, we tested whether CsMPK6 affects the transactivation activity of CsMYC2. We coexpressed different forms of CsMYC2 (i.e. CsMYC2, CsMYC2D, CsMYC2A) as GFP fusion proteins or we coexpressed CsMYC2-GFP and CsMPK6-myc (CsMYC2/CsMPK6) with promoters targeted by CsMYC2 fused to the LUC reporter gene in *N. benthamiana* leaves or “Newhall” citrus calli and performed LUC activity assays. CsMYC2A had the highest transactivation activity on the *CsCCD4b* promoter. In contrast, CsMYC2D only slightly induced expression from the *CsCCD4b* promoter. The coexpression of CsMYC2 and CsMPK6 reduced the transactivation activity of CsMYC2 on the *CsCCD4b* promoter (Figure 5C; Supplemental Figure 8A). A MeJA treatment enhanced the transactivation activity of CsMYC2 on the *CsCCD4b* promoter (Figure 5C; Supplemental Figure 8A). We also tested whether CsMPK6 regulates the activities of the *CsPSY*, *CsLCYb*, *CsBCH*, and *CsMPK6* promoters using a LUC reporter gene activity assay and obtained similar results (Supplemental Figures 8 and 14). Protein–protein interactions and phosphorylation often alter the locations of TFs. However, CsMYC2-GFP accumulated in the nucleus regardless of whether we coexpressed CsMYC2-GFP and CsMPK6-myc in *N. benthamiana* leaves (Supplemental Figure 15). These results indicated that CsMPK6 attenuates the transactivation activity of CsMYC2 by inhibiting its DNA-binding activity and by phosphorylating CsMYC2 without affecting its subcellular localization.

### CsMYC2 and CsMPK6 antagonistically regulate the accumulation of β-citraurin in citrus

**Figure 6:**
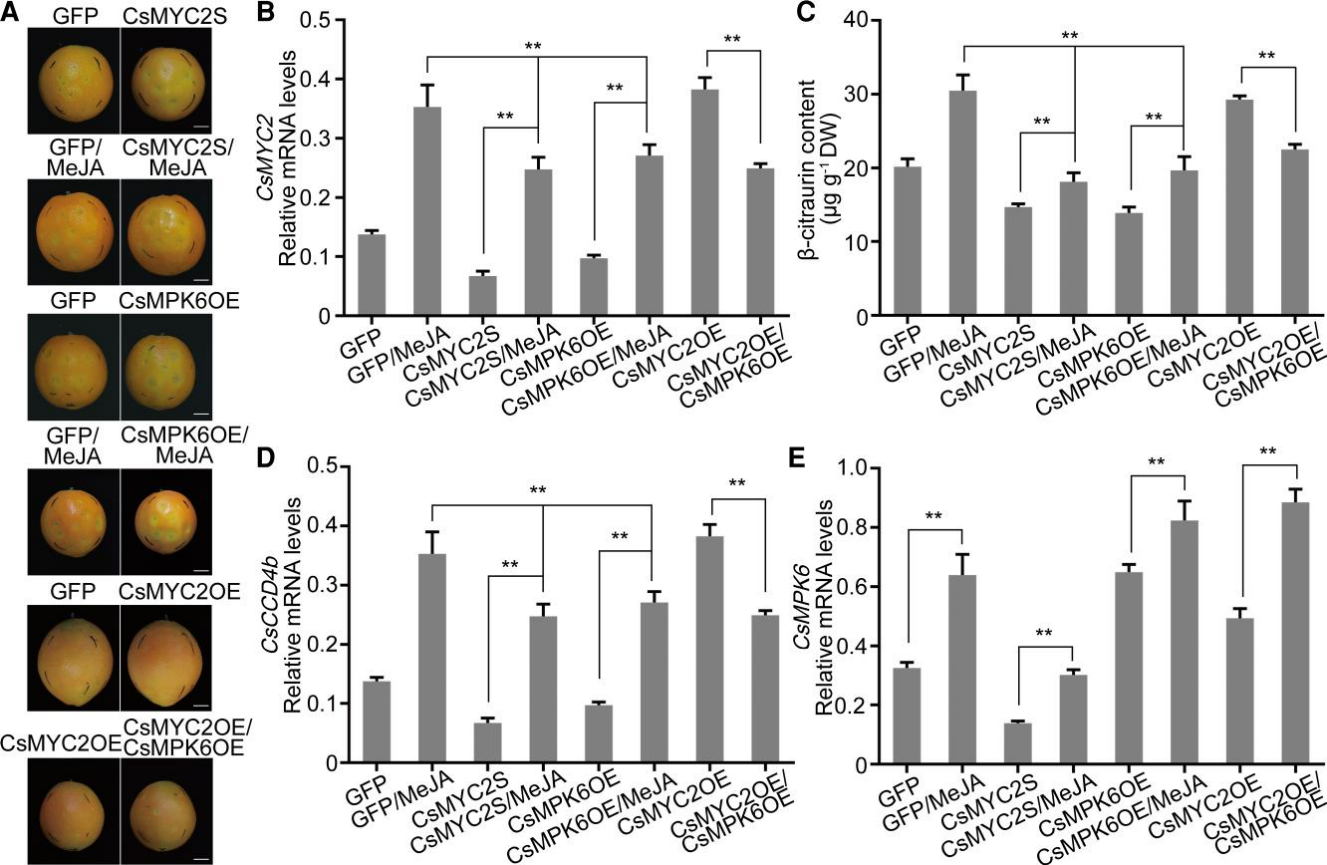
Attenuation of CsMYC2-induced β-citraurin biosynthesis by CsMPK6. A–E, Silencing and overexpression of CsMYC2 (CsMYC2S, CsMYC2OE) and overexpression of CsMPK6 (CsMPK6OE) in on-tree citrus fruit at 195 DAFB in 2021 using *Agrobacterium*-mediated transient transformation (A, phenotypes). MeJA treatment was performed 3 d after infiltration. Fruit was harvested 15 d after infiltration. Fruit overexpressing GFP was used as a control. Scale bars, 1 cm. B, *CsMYC2* mRNA levels evaluated in infiltrated fruit using RT-qPCR to ensure successful infection. The β-citraurin content was determined C, and the mRNA levels of *CsCCD4b* D, and *CsMPK6* E, were measured in infiltrated fruit using RT-qPCR. Peel samples from each group of infiltrated fruits were divided into three sets. Each set contained peels from five fruits. An independent RNA or carotenoid extraction from each set of peels was used as one biological replicate. Three replicates were performed. Values represent means ± SE. Asterisks indicate statistically significant differences, as determined by a Student's *t* test (**P < .01). n.s., no significant difference.

We explored the roles of CsMYC2 and CsMPK6 in fruit coloration using transient expression assays. We overexpressed the CDS from *CsMPK6* as a GFP fusion protein with an overexpression vector (CsMPK6OE) and used the *35S* promoter in pRI101 to drive the expression of a partial antisense transcript of *CsMYC2* (i.e. to generate a *CsMYC2* silencing vector that we named CsMYC2S). We introduced the CsMYC2-GFP (CsMYC2OE), CsMPK6-GFP, and CsMYC2S vectors into *Agrobacterium* and infiltrated the peels of different pieces of fruit on a tree with these cultures. Three d after infiltration, we sprayed the fruit with MeJA and harvested the fruit 15 d after infiltration. The empty GFP overexpression vector was used as a control (Figure 6A; Supplemental Figure 16A). The expression of *CsMYC2* was significantly downregulated in CsMYC2S citrus fruit peels relative to the control (Figure 6B). The accumulation of β-citraurin and fruit coloration were also reduced (Figures 6, A and C; Supplemental Figure 16A).

When the fruits were sprayed with MeJA, the area of the fruit subjected to *CsMYC2* silencing appeared pale and contained significantly lower levels of β-citraurin relative to the control (Figures 6, A and C; Supplemental Figure 16A). The expression patterns of *CsCCD4b* and *CsMYC2* was similar (Figure 6D). Similarly, in *CsMPK6-*overexpressing fruit, the expression levels of both *CsMYC2* and *CsCCD4b* were reduced (Figure 6, B, D, and E), and the infiltrated area on the fruit was pale, with lower β-citraurin concentrations than the control (Figures 6, A and C; Supplemental Figure 16A). These changes were partially rescued by a treatment with exogenous MeJA (Figure 6, A–D; Supplemental Figure 16A). In contrast, the overexpression of *CsMYC2* led to stronger *CsCCD4b* expression, β-citraurin accumulation, and red coloration in peels compared to the controls (Figure 6, A–D; Supplemental Figure 16A). When *CsMYC2* was coexpressed with *CsMPK6*, less red coloration was detected in the infiltrated areas, and the expression of *CsCCD4b* and β-citraurin content were reduced (Figure 6, A, C, D, and E; Supplemental Figure 16A). Finally, we examined the expression of *CsPSY*, *CsLCYb*, and *CsBCH* and the concentrations of β-carotene, β-cryptoxanthin, and violaxanthin. We found that they all shared similar patterns of β-citraurin accumulation (Supplemental Figure 16). These results indicate that CsMYC2 is indispensable for JA-induced citrus fruit coloration and that CsMPK6 downregulates this process.

## Discussion

Carotenoids are important natural pigments that determine fruit color in plants such as tomato, apricot (*Prunus armeniaca*), durian (*Durio zibethinus*), and citrus (Marty et al., 2005; Matsumoto et al., 2009; Nisar et al., 2015; Wisutiamonkul et al., 2017). To date, although numerous studies have revealed that many different mechanisms regulate carotenoid biosynthesis, the mechanism used by JA to regulate carotenoid biosynthesis during color development in citrus was unknown. Here, we demonstrated that MeJA treatment promotes β-citraurin biosynthesis and fruit coloration in “Newhall” orange by activating the TF CsMYC2, which in turn binds the promoters of β-citraurin biosynthetic genes (*CsCCD4b*, *CsPSY*, *CsLCYb*, *CsBCH*) and induces their expression. Meanwhile, CsMYC2 activates the transcription of *CsMPK6*, and CsMPK6 reduces the DNA-binding activity and stability of CsMYC2 by interacting with CsMYC2. Thus, CsMPK6 serves as a negative feedback regulator that attenuates the response of citrus to JA. Our findings provide detailed information on the involvement of JA in the biosynthesis of the carotenoid β-citraurin and fruit coloration in citrus.

In tomato, elevated levels of endogenous jasmonates and jasmonate signaling significantly increased lycopene accumulation and fruit coloration (Liu et al., 2012). In contrast, tomato fruit accumulated little lycopene when the biosynthesis of endogenous JA was impaired (Tzortzakis and Economakis, 2010; Liu et al., 2012). In citrus, the endogenous JA content was reported to fluctuate with a “decrease–increase–decrease” pattern during fruit development and maturation (Feng et al., 2021). In the current study, we observed that endogenous JA and JA-Ile concentrations increased during fruit development and maturation, peaked at 210 DAFB, and decreased at 240 DAFB in “Newhall” orange (Supplemental Figure 1, A and B). These findings are somewhat consistent with previous findings. The patterns of β-citraurin accumulation and *CsCCD4b* expression were similar relative to the accumulation of endogenous JA (Supplemental Figure 1). Therefore, we speculate that, similar to the influence of JA on the accumulation of lycopene in tomato, increases in endogenous levels of JA may contribute to the accumulation of β-citraurin during fruit development and maturation in orange. However, although we showed that exogenous MeJA treatments promoted endogenous jasmonates accumulation and β-citraurin biosynthesis, and thus fruit coloration in citrus fruits, experiments with mutants that are deficient in JA and that over-accumulate JA are required to determine whether endogenous JA is indispensable for the accumulation of β-citraurin. Although JA-Ile levels peaked at 210 DAFB during fruit development and maturation, we observed a continuous increase in JA-Ile in the control fruit that was harvested at 210 DAFB (Supplemental Figure 3C), therefore, the physiological range of JA-Ile during the fruit maturation process in citrus fruit still needs to be determined with a thorough analysis of citrus fruit with a detailed analysis during the on-tree maturation process.

Ethylene and ABA are believed to influence carotenoid biosynthesis. Recently JA was demonstrated to directly regulate carotenoid biosynthesis (Liu et al., 2015). In tomato, an exogenous MeJA treatment significantly stimulated the accumulation of lycopene and fruit coloration in the fruit of the ethylene insensitive mutant *Nr* (Liu et al., 2012). Similarly, the application of MeJA accelerated the coloration of fruit from tomato and mandarin that were treated with 1-MCP (i.e. fruit with attenuated ethylene signaling) (Gómez et al., 2017; Rivero Meza et al., 2021). These authors proposed that JA uses an ethylene-independent mechanism to promote fruit coloration. In this study, we observed that a MeJA treatment promoted fruit coloration in orange and mandarin fruits without affecting ACC content (Supplemental Figure 4). Although the application of MeJA stimulated the accumulation of ABA, we think that the MeJA-induced increases in carotenoid biosynthesis explain these increases in ABA because carotenoids are precursors for ABA biosynthesis (Supplemental Figures 3 and 4). Consistent with this interpretation, we found that when ABA biosynthesis was disrupted by an NDGA treatment, MeJA no longer induced increases in the levels of ABA and that MeJA treatments still promoted fruit coloration (Supplemental Figure 4). These results indicate that ethylene and ABA are not required for JA to promote fruit coloration. Therefore, we conclude that at least to some extent, JA uses an ethylene- and ABA-independent mechanism to directly promote the accumulation of β-citraurin and fruit coloration. However, the mechanisms that regulate the accumulation of β-citraurin are poorly understood. For example, the influence of ethylene, ABA, and other signals that regulate β-citraurin biosynthesis remains poorly understood. Thus, identifying central regulatory mechanisms and testing whether JA influences these mechanisms during β-citraurin biosynthesis and fruit coloration are important tasks for further study.

The expression levels of β-citraurin biosynthetic genes (*CsCCD4b*, *CsPSY*, *CsLCYb*, *CsBCH*) and *CsMYC2* continued to decline after the MeJA treatment (Figures 1 and 2A), which indicates that the response of citrus to JA was attenuated. We reasoned that inhibitor(s) of JA signaling might have been activated in the fruit. PUBs, BPMs, and MPKs are important suppressors of JA signaling that probably interact with MYCs (Zhai et al., 2013; Jung et al., 2015; Chico et al., 2020). Indeed, we observed that CsMPK6 interacted with CsMYC2 (Figures 3, A–C) and that the presence of CsMPK6 significantly reduced the transactivation activity of CsMYC2 on particular gene promoters (Figure 5C; Supplemental Figures 8 and 14). This observation can be partially explained by the notion that MPK6 contributes to the negative feedback regulation of JA signaling by phosphorylating MYC2, which leads to its proteolysis, in *Arabidopsis thaliana* (Zhai et al., 2013; Sethi et al., 2014). These data resemble our findings (Figure 4, A, C, E, F, and H). Our finding that the promoter activities of CsMYC2 target genes were lower in the presence of CsMYC2D and higher in the presence of CsMYC2A, further support this interpretation (Figure 5C; Supplemental Figures 8 and 14).

However, although the DNA-binding activities of CsMYC2, CsMYC2D, and CsMYC2A were not significantly different (Figure 5B; Supplemental Figure 13B) and the subcellular distribution of CsMYC2 was not affected by CsMPK6 (Supplemental Figure 15), interactions between CsMYC2 and CsMPK6 inhibited the promoter-binding activity of CsMYC2 (Figure 5A; Supplemental Figure 13A). This finding could also explain the inhibition of JA signaling by CsMPK6. Collectively, these results indicate that CsMPK6 uses two mechanisms to inhibit JA signaling. CsMPK6 decreases the stability of the CsMYC2 protein by phosphorylating CsMYC2 and decreases the DNA-binding activity of CsMYC2 by binding CsMYC2. Since JA is a stress-responsive plant hormone, a long-term, constant JA response may be harmful for plant development (Kazan and Manners, 2013; Zhai et al., 2013; Song et al., 2014; Wasternack and Song, 2017; Aerts et al., 2020). Therefore, we propose that this “phosphorylation for degradation” and “inhibition of binding” double-track negative feedback loop is an important process that balances JA signaling during citrus coloration and that CsMPK6 is critical for preventing citrus fruit from overreacting to JA. Moreover, we found that *CsMYC2* expression was significantly reduced in citrus that overexpressed *CsMPK6* (Figure 6B), which was also reported in Arabidopsis (Takahashi et al., 2007). However, the underlying mechanism remains unclear.

MYC TFs are master regulators of JA signal transduction (Kazan and Manners, 2013). The phosphorylation of T328 on MYC2 by MPK6 is important for inducing both its transcriptional activity and proteolysis in Arabidopsis during the immune response (Zhai et al., 2013). However, MYC2 is activated by MPK6-mediated phosphorylation at S123, which does not induce its proteolysis during the developmental response of Arabidopsis seedlings to blue light (Sethi et al., 2014). We identified eight sites (T236, T325, S326, T334, S335, S339, S485, and S486) on CsMYC2 that are phosphorylated by CsMPK6. This difference between citrus and Arabidopsis indicates that the sites on MYC2 that are phosphorylated by MPK6 differ among species or biological processes. In addition, although the degradation of CsMYC2 was also significantly accelerated by its phosphorylation (Figure 4F), in contrast to Arabidopsis, the activities of the promoters from *CsCCD4b*, *CsPSY*, *CsLCYb*, *CsBCH*, and *CsMPK6* were significantly induced by CsMYC2A (i.e. the nonphosphorylatable form of CsMYC2) (Figure 5C; Supplemental Figures 8 and 14). Therefore, these data are consistent with the phosphorylation of CsMYC2 substantially influencing protein stability rather its transcriptional activity in citrus. We examined the relationships between CsMYC2 and both CsPUBs and CsBPMs because either might contribute to the degradation of CsMYC2, but no interactions were observed (Supplemental Figure 10). Thus, an important task for the future is to identify the protein that induces the proteolysis of CsMYC2.

**Figure 7:**
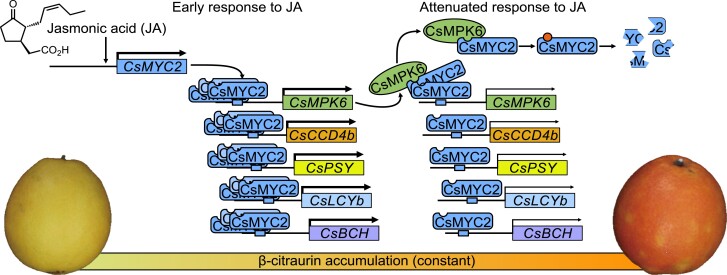
JA-activated CsMYC2 binds the promoters of CsCCD4b and other β-citraurin biosynthetic genes and induces their expression to enhance the coloration of ‘Newhall’ orange fruit. During this early JA response process, the transcription of the CsMPK6gene is activated by CsMYC2. Additionally, the CsMPK6 protein binds the CsMYC2 protein and accelerates the degradation of CsMYC2. This negative feedback regulation attenuates both the promoter binding activity of CsMYC2 and the response of citrus fruit to JA.

In conclusion, JA promotes fruit coloration in “Newhall” orange mainly by upregulating the expression of *CsMYC2* expression, which induces the expression of genes required for the biosynthesis of β-citraurin. CsMYC2 also induces the expression of *CsMPK6* (Figure 7A). In turn, CsMPK6 interacts with CsMYC2 and inhibits the promoter-binding activity of CsMYC2. CsMPK6 also phosphorylates CsMYC2, which decreases the stability of CsMYC2 and thus, attenuates the response of citrus to JA (Figure 7B). Our findings demonstrate the molecular details of a JA signaling mechanism and a negative feedback loop that regulates the accumulation of carotenoids and fruit coloration in citrus.

## Materials and methods

### Plant material and treatments

Navel orange (*Citrus sinensis* cv. Newhall) fruits were collected from the Huazhong Agricultural University (Wuhan, China) experimental farm in 2020. Fruits were grown under natural light and harvested every 30 d beginning 120 DAFB (days after full blossom) and ending 240 DAFB. At each time point, 15 orange fruits were harvested from 10 trees and divided into five sets that contained three fruits per set. The peels (i.e. the epicarp, without the albedo) from each set of fruit were removed using surgical blades and immediately quick-frozen using liquid nitrogen. The peel samples from each set of fruits were separately and evenly mixed and then stored at −80°C for plant hormone, carotenoid, and gene expression analyses. Each set of peels was used as one biological replicate. For on-tree treatments, the fruits were sprayed with 0.2, 0.5, 1, and 4 mM MeJA (catalog no. 392707, Sigma-Aldrich, USA) at 180 DAFB. Untreated fruits were used as controls. Five fruits were used for each treatment. One fruit was used as one biological replicate. Fruits were photographed and collected at 200 DAFB. For JA inhibitor treatments, the on-tree fruits were sprayed with 5 mM sodium diethyldithiocarbamate (DIECA, catalog no. SD3506, Real-Times Biotechnology, Beijing, China) at 160 DAFB in 2022. Untreated fruits were used as a control. The treatment was repeated every 5 d. Fifteen fruits were used for each treatment. One fruit was used as one biological replicate. Fruits were collected at 180 DAFB. For off-tree treatments, similarly colored orange fruits (approximately 140 fruits) were harvested from twenty trees at 210 DAFB and immediately transferred to the laboratory for treatment. Fruits were separated into two groups that contained 70 fruit per group. The first group was submerged in 0.5 mM MeJA for 2 h. The second group did not receive any treatment and was used as the control. All fruit was stored at room temperature for 20 d. The fruit peels (i.e. the epicarps) were sampled every 5 d. The day of harvest was defined as 0 DAT. At each sampling time point, nine fruits from each treatment were divided into three sets that contained three fruits per set. Peels from each set of fruit were sampled and immediately frozen using liquid nitrogen, evenly mixed and stored at −80°C for experiments. Each set of peels was used as one biological replicate.

Lane Late Navel orange (*Citrus sinensis* L. Osbeck) fruits were collected from the Citrus Variety Propagation Centre (Zigui County, Yichang, Hubei, China) in 2022. Similar-colored fruits (approximately 180 fruits) were harvested at 400 DAFB from thirty trees and immediately transferred to the laboratory. Fruits were divided into five groups. Each group contained 36 fruits. The first group did not receive any treatment and was used as a control. The second group was treated with 5 mg L^−1^ 1-methylcylopropene (1-MCP), an ethylene inhibitor (catalog no. M875517, Macklin, Shanghai, China), in an airtight box for 12 h as described by Sun et al. (2021). The third group was first treated with 1-MCP and subsequently treated with MeJA, as described above. The fourth group was submerged in 0.5 mM NDGA an ABA inhibitor (catalog no. IN590, Solarbio Life Science, Beijing, China) for 2 h as described by Wang et al. (2016a). The fifth group was treated with NDGA, stored at room temperature for 10 h, and subsequently treated with MeJA as described above. All fruits were stored at room temperature for 14 d and photographed every 7 d. The day of harvest was defined as 0 DAT.

Orah mandarin (*Citrus reticulata* Blanco) fruits were collected from the Guangxi Academy of Specialty Crops (Guilin, Guangxi, China) in 2021. Fruits were harvested at 210 DAFB from 20 trees and immediately transferred to a cold storage warehouse and stored at 4°C for 210 d. Before treatment, similar-colored fruits (approximately 100 fruits) were selected and transferred to the laboratory. After a 3 h recovery period that allowed the fruit temperature to equilibrate to room temperature, the fruits were divided into five groups that each contained 20 fruits. The treatment regime was as described for the Late Lane Navel orange. All fruits were stored at room temperature for 7 d and photographed at 7 DAT. The treatment day was defined as 0 DAT.

### Quantification of carotenoid content

Orange peel samples were lyophilized using a lyophilizer (catalog no. 7960070; LABCONCO FreeZone, USA). Carotenoid extractions were performed as previously described by Zhu et al. (2021b) and Zheng et al. (2019). The extracts were analyzed using high-performance liquid chromatography (e2695; Waters, USA) as previously described (Zheng et al., 2019). The carotenoids were identified by comparing their retention times with the retention times of authentic standards (β-carotene, catalog no. CTN0003; β-cryptoxanthin, catalog no. 072624203780617; β-citraurin, catalog no. CTN0483; violaxanthin, catalog no. 228915278393478, CaroteNature, Lupsingen, Switzerland). The authentic standards were used to construct standard curves for determining carotenoid concentrations by integrating peak areas. The levels of each carotenoid were quantified by converting the pertinent peak area to a concentration using the appropriate standard curve. An independent extraction from each set of fruit peels was used as one biological replicate. At least three biological replicates from independent extractions for three sets of fruit peels were performed.

### RNA extraction and RT-qPCR

RNA extractions and cDNA synthesis were performed as previously described (Li et al., 2017b). Gene mRNA levels were quantified using quantitative RT-qPCR with an ABI 7,500 real-time system. The reaction system and program were described by Yue et al. (2020) and Ji et al. (2022). The expression of *ACTIN* (XM_006486038) was used as an internal control. The peels from nine fruits sampled at each time point were divided into three groups that contained three fruits per group. The peels from each group were evenly mixed for RNA extraction. RNA extracted from each group was used as one biological replicate, and three replications were analyzed. For “Newhall” calli, each line of infected calli was used as one biological replicate. Three lines from independent infections were used in each experiment. All primers are shown in Supplemental Data Set 1.

### *Agrobacterium*-mediated infiltration and infection

The CDS of *CsMYC2* (CsMYC2-GFP, CsMYC2OE) was ligated into pRI101-GFP to make plasmids that express green fluorescent protein (GFP) fusion proteins. This vector was constructed by inserting a GFP tag upstream of the multiple cloning site in pRI101. The CDS of *CsMPK6* was inserted into both pRI101-GFP and pCAMBIA1305-myc to generate the overexpression constructs CsMPK6-GFP and CsMPK6-myc. To silence the expression of *CsMYC2*, a partial CDS of *CsMYC2* (611–961 bp, CsMYC2S) was ligated into pRI101 to express a partial antisense transcript from *CsMYC2* using a Seamless Cloning Kit (catalog no. D7010M; Beyotime, Shanghai, China). These constructs were introduced into *Agrobacterium tumefaciens* (strain EHA105) and cultured as described in Li et al. (2016). Preparation of the *Agrobacterium* suspension and on-tree citrus infiltration experiments were performed as described by Gong et al. (2021) and Yue et al. (2020) with minor modifications. Briefly, a needle was used to generate 0.5 mm deep pinholes on two sides of a fruit (4–6 pinholes per side). The pinholes on both sides were gently infiltrated with approximately 100–200 *μ*L of an *Agrobacterium* suspension with a 1 mL needleless syringe. For each fruit, the side infiltrated with empty pRI101-GFP was used as a negative control. CsMYC2OE was used as a control for fruit coexpressing CsMYC2 and CsMPK6. The infiltrated fruit was divided into two groups: one group received no treatment. The other group was sprayed with 1 mM MeJA 3 d after infiltration. All fruits were collected 15 d after infiltration. The infiltrated area of the peel was collected and divided into three groups to quantify gene expression and carotenoid content. RNA extractions and carotenoid extractions were performed for each group and used as one biological replicate. Three biological replicates were performed.

The “Newhall” callus was induced from undeveloped ovules that were collected from “Newhall” orange fruits from 200 to 220 DAFB in the dark at room temperature on induction medium, which is MT medium containing 8 g L^−1^ agar, 40 g L^−1^ sucrose and 0.1 mg L^−1^ IAA. The resulting callus was then allowed to proliferate on MT solid medium containing 8 g L^−1^ agar and 40 g L^−1^ sucrose. For the infection of “Newhall” callus, *A. tumefaciens* strain EHA105 harboring various vectors was resuspended in MT medium (catalog no. LA0120; Solarbio Life Science, Beijing, China) containing 0.1 mM acetosyringone. The calli were soaked in the *Agrobacterium* culture for 20 min and collected using a cell strainer (catalog no. CSS010040; Jet Biofil, Guangzhou, China). The infected calli were incubated on MT solid medium for 3 d. To treat the calli with JA, 50 *μ*M MeJA was sprayed on calli 2 h before performing each experiment. Each individual infection of citrus callus was used as one replicate. Three replicates were performed. The primers used in these experiments are listed in Supplemental Data Set 1.

### ChIP-PCR assay

**‘**Newhall” callus proliferated for 2 weeks and was evenly divided into two groups. The first group was used for the high-level expression of GFP and served as a negative control. Another group was used for the high-level expression of CsMYC2-GFP. Each group contained six sets of calli. Three sets of calli in each group were selected and subjected to a MeJA treatment. Each set of calli was defined as one infected line and used as one biological replicate. Three infected lines were used for ChIP-PCR experiments as three biological replicates. All calli were infected together at the same time, the infection procedure and MeJA treatment are described above. Calli overexpressing either CsMYC2-GFP or GFP were subjected to ChIP-PCR as described in Li et al. (2017b). The ChIP experiment was performed using a SimpleChip Plus Sonication Chromatin IP Kit (catalog no. 56383; Cell Signaling Technology, Danvers, MA, USA) following the manufacturer's instructions. The chromatin was fragmented using a Vibra-cell VCX 150 sonicator (Sonics & Materials Inc.; Newtown, CT, USA) for 5 s followed by 8 s without sonication for 21 cycles. Fragmented chromatin (7 *μ*g) was used for immunoprecipitation experiments that utilized an anti-GFP antibody (0.4 mg mL^−1^, catalog no. 11814460001; Roche, New York, NY, USA). The enriched and fragmented chromatin immunoprecipitated from each line was used as one biological replicate and was analyzed using qPCR. DNA from three lines was used as three biological replicates. The ChIP results were quantified as the % input as described by Haring et al. (2007). The enrichment from the negative control expressing only GFP was defined as 1. The final ChIP results were represented as the fold enrichment relative to the GFP negative control. The ChIP-PCR results were quantified as the average of three biological replicates. Four promoter regions from *CsCCD4b*, three from *CsPSY*, four from *CsLCYb*, four from *CsBCH*, and four from *CsMPK6* were amplified using PCR with specific primers (Supplemental Table 1) to quantify their enrichment. The length of each region was approximately 200 bp.

### Dual-luciferase activity assays

The numbering of bp in the promoters is defined relative to the translation start site, which is defined as +1. The promoters from *CsCCD4b* (beginning at −1,800 relative to the translation start site, TSS), *CsPSY* (beginning at −1,488 relative to the TSS), *CsLCYb* (beginning at −1,400 relative to the TSS), *CsBCH* (beginning at −1,400 relative to the TSS), and *CsMPK6* (beginning at −1,300 bp relative to the TSS) were separately introduced into the pGREENII 0800-LUC reporter vector. *Agrobacterium* strain EHA105 was separately transformed with each construct. The CsMYC2 and CsMPK6 overexpression vectors were used as effectors. Strains harboring the effectors and reporters were coinfiltrated into *Nicotiana benthamiana* leaves. The plants were incubated at room temperature for 3 d. The LUC activity assay was performed according to the manufacturer's instructions for the DUAL-GLO Luciferase assay system (catalog no. E2920; Promega, Madison, WI, USA). MeJA (10 *μ*M) was infiltrated into *N. benthamiana* leaves 1 h before the assessment. For LUC activity assays in “Newhall” callus, the infection procedure was performed as described above, and LUC activity was quantified as described for *Nicotiana benthamiana.* The primers are listed in Supplemental Data Set 1.

### CoIP assay

*N. benthamiana* leaves were coinfiltrated with *Agrobacterium* strain EHA105 harboring CsMYC2-GFP and CsMPK6-myc. Leaves infiltrated with empty pRI101-GFP and CsMPK6-myc were used as negative controls. For the CoIP assay, the leaves were quick-frozen in liquid nitrogen, ground into a powder, incubated in 500 *μ*L of PE buffer (50 mM Tris-MES, pH 8, 0.5 M sucrose, 1 mM MgCl_2_, 10 mM EDTA, pH 8, 5 mM DTT, and 1 mM PMSF) on ice for 30 min, and subjected to high-speed centrifugation for 30 min to obtain the supernatant (i.e. the whole-leaf extract). The supernatant was incubated with anti-GFP magnetic beads (catalog no. D153-11, MBL, Beijing, China) to immunoprecipitate either CsMYC2-GFP or GFP. The immunoprecipitate was analyzed by immunoblotting with an anti-Myc antibody (1 mg mL^−1^, catalog no. 2276; Cell Signaling Technology, Danvers, MA, USA).

### Pull-down and protein phosphorylation assays

The CDSs of *CsMYC2* and *CsMPK6* were separately ligated into pMAL-C2X and pGEX4T-1 for the expression of fusion proteins in *E. coli*. The CDSs for the *N*-terminus (amino acids 1–250) and the C-terminus (amino acids 251–519) of *CsMYC2* according to the NCBI Conserved Domain analysis tool (www.ncbi.nlm.nih.gov/Structure/cdd/wrpsb.cgi) was ligated into pMAL-C2X. Protein purification was performed as described by Li et al. (2017b). The CsMYC2-MBP fusion protein was bound to MBP magnetic beads (catalog no. E8035S; New England Biolabs, MA, USA) and incubated with CsMPK6-GST on ice for 1 h. Subsequent steps are described by Li et al. (2017b). The GST protein was used as a negative control.

The CDSs from *CsMYC2* and *MKK4DD* were separately ligated into pGEX4T-1 and pET30A^+^ for the expression of proteins in *E. coli*. For the in vitro phosphorylation reaction, CsMPK6-GST (20 *μ*g) was activated by incubation with MKK4DD-His (2 *μ*g) in reaction buffer (40 mM HEPES, pH 7.5, 20 mM MgCl_2_, 1 mM DTT, 2 mM ATP) at 37°C for 2 h. CsMYC2-GST (5 *μ*g) was then phosphorylated by activated CsMPK6-GST in the same reaction buffer at 37°C for 2 h. The reaction was stopped by adding SDS gel-loading buffer followed with vigorous mixing. Phosphorylation was detected using Phos-bind Biotin BTL-104 (APE BIO, Houston, TX, USA) according to the manufacturer's instructions. The phosphorylation sites on CsMYC2-GST and CsMPK6-GST were identified using a LC/MS analysis that was performed by Novogene Company (www.novogene.com). For the in vivo phosphorylation assay, the CDS from *CsMPK6D* (Y225 to D225) was ligated into pCAMBIA1305-myc and co-overexpressed with CsMYC2-GFP in *N. benthamiana* leaves. Total protein and CsMYC2-GFP extraction were performed as described above, and phospho-signals were detected. *N. benthamiana* overexpressing only CsMYC2-GFP was used as a control. The sequences of *MKK4DD* according to Wang et al. (2016b) and *CsMPK6D* were synthesized by Tsingke Company (tsingke.com.cn). The primers used in these experiments are listed in Supplemental Data Set 1.

### Luciferase complementation experiment

The CDSs from *CsMYC2* and *CsMPK6* were separately ligated into the JW-771-nLUC and JW-772-cLUC vectors, introduced into *Agrobacterium* strain EHA105 and expressed in *N. benthamiana* leaves. Chemiluminescence produced by LUC was observed as described by Li et al. (2017b) on a NightSHADE LB 985 imaging system (Berthold Technologies, Germany). MeJA treatment was performed as described above. The primers are listed in Supplemental Data Set 1.

### Protein degradation assay

The in vitro cell-free degradation assay was performed as described by He et al. (2020) with minor modifications. Briefly, total proteins were isolated from 5 DAT samples harvested at 210 DAFB and treated with MeJA, evenly mixed, equally dispersed into reaction tubes containing CsMYC2-MBP (2 *μ*g) and GST (1 *μ*g) or CsMYC2-MBP (2 *μ*g) and CsMPK6-GST (1 *μ*g) and incubated at 37°C for the indicated periods of time.

For the in vivo CsMYC2 accumulation assay, the CDS of *CsMYC2D* (all phosphorylation sites changed to D) and *CsMYC2A* (all phosphorylation sites changed to A) were ligated into pRI101-GFP and introduced into *Agrobacterium* strain EHA105. CsMYC2-GFP, CsMYC2D-GFP, CsMYC2A-GFP, or both CsMYC2-GFP and CsMPK6-myc were transiently expressed in “Newhall” calli and were collected 3 d after infection. The *CsMYC2D* and *CsMYC2A* sequences were synthesized by Tsingke Company. Total protein was isolated as described above and analyzed by immunoblotting using an anti-GFP antibody.

For the in vivo CsMYC2 degradation experiment, CsMYC2-GFP-expressing calli (CsMYC2-GFP) were treated with MeJA for 30 min, transferred to new MT solid medium containing 50 *μ*M CHX, and incubated at room temperature for the indicated periods of time. Calli cultured on a medium containing both 50 *μ*M cycloheximide (CHX) (catalog no. 5.08739; Merck, USA) and 50 *μ*M MG132, a proteasome inhibitor (catalog no. SML1135; Sigma-Aldrich, USA), were harvested at 8 h. CsMYC2-GFP calli treated with DMSO (CK) were used as a control. All transient expression assays were performed at least three times. Total protein was extracted as described above and was analyzed using immunoblotting with an anti-GFP antibody. The primers used in these experiments are listed in Supplemental Data Set 1.

### DNA pull-down assay

The biotin-labeled promoter probe from *CsCCD4b* (Supplemental Figure 7) was synthesized by Sangon Company (www.sangon.com). The probe was incubated with streptavidin-conjugated magnetic beads (catalog no. 22307-1; Beaver Bio, Suzhou, China) in binding buffer (10 mM Tris-HCl, pH 7.5, 1 mM EDTA, 1 M NaCl, 0.1% Tween-20) at room temperature for 30 min. The premixed CsMYC2-MBP and CsMPK6-GST proteins were incubated with probe-bond beads for 1 h on ice. The beads were collected and washed well. SDS gel-loading buffer was added to the probe-protein complex and was analyzed using immunoblotting with anti-MBP or anti-GST antibodies (1,000 *μ*g ml^−1^, catalog no. HRP-66001; Proteintech, Rosemont, IL, USA). Probe-bound beads incubated with premixed CsMYC2-MBP and GST, CsMYC2-MBP, only GST or CsMPK6-GST were used as controls. To investigate the binding activity of CsMYC2, the CDSs from *CsMYC2D* and *CsMYC2A* were ligated into the pMAL-C2X vector. MBP fusion proteins were expressed in *E. coli* and purified. The experiment was performed as described above. MBP was used as a negative control. The primers used in these experiments are listed in Supplemental Data Set 1.

### Quantification of endogenous plant hormones

For endogenous JA, JA-Ile, OPDA, ACC, and ABA measurements, the orange peel samples (0.5 g) were extracted with 2 mL of extraction solution (isopropanol: distilled water: concentrated HCl, 2:1:0.002, v/v/v) that contained 10 ng of H_2_JA (dihydrojasmonic acid, catalog no. 0145324, Olchemim, Czech Republic) and 10 ng of d_6_-ABA (catalog no. ID1001, Icon Isotopes), and incubated on an orbital shaker at 180 rpm for 1 h at 4°C. Dichloromethane (4 mL) was added. Then, the mixtures were shaken at 180 rpm for 1 h. The mixtures were centrifuged at 10,000 × *g* at 4°C for 10 min. The lower phase of the extract was collected and transferred to a new tube and then concentrated and dried under a stream of nitrogen. The concentrated plant hormone extract was then dissolved in 200 *μ*L of methanol and analyzed using the UHPLC/MS system (Ultimate 3000 TSQ Altis, ThermoFisher Scientific, USA). H_2_JA and d_6_-ABA were used as internal standards for quantifying JA, JA-Ile, OPDA, and ABA levels. Different concentrations of ACC (catalog no. A3903, Sigma-Aldrich, USA) were used as external standards for the construction of a standard curve based on integrated peak areas. Endogenous ACC levels were quantified using a standard curve to convert peak areas to concentrations. An independent extraction from each set of fruit peels was used as one biological replicate. At least three biological replicates from independent extractions for three sets of fruit peels were analyzed.

### Subcellular localization analysis

NF-YA4-mCherry was used as a nuclear marker (Yue et al., 2020) and was co-overexpressed with either CsMYC2-GFP or GFP in *N. benthamiana* leaves, followed by an incubation at room temperature for 3 d. Fluorescence was observed with a confocal microscope (TCS SP8, Leica, Germany). All transient expression experiments were performed at least three times.

### Yeast one-hybrid assay

The CDS from *CsMYC2* was inserted into pGADT7. The *CsCCD4b* promoter was ligated into pABAi. A Y1H assay was performed as described previously (Yue et al., 2020). The primers used in these experiments are listed in Supplemental Data Set 1.

### Yeast two-hybrid assay

The CDS of *CsMPK6* was introduced into pGADT7. The CDSs from *MKK4DD*, *PUBs*, and *BPMs* were separately inserted into pGBKT7. Y2H assays were performed to test for interactions between CsMYC2 and PUBs, BPMs, CsMPK6, and MKK4DD as described by Li et al. (2017b). The primers used in these experiments are listed in Supplemental Data Set 1.

### Electrophoretic mobility shift assay

The CsMYC2-MBP fusion protein was purified. The 3′ biotin-labeled probes were synthesized by Sangon Company. The sequences are shown in Supplemental Figure 6. EMSAs were performed as previously reported (Yue et al., 2019). MBP was used as a negative control.

### Biolayer interferometry assay

The CDS from *CsMYC2* was inserted into the pET30A protein expression vector. The CsMYC2-His protein was expressed in *E. coli* and purified. The BLI assay was conducted on an Octet RED 96 System with SA sensors (Forte Bio, Menlo Park, CA, USA). The sensors were soaked with PBS (catalog no. CW0040, CWBio, Beijing, China) for 5–10 min and then equilibrated for 60 s and associated with 100 nM biotin-labeled *CsCCD4b* promoter probe that was also used in the EMSA experiment for 120 s. After a 60 s re-equilibration, consequent sensors were soaked with different concentrations of CsMYC2-His protein (1000, 500, 250, 125, 62.5 nM) which was diluted in PBS and served as an analyte for 120 s. Then soaked in PBS to permit disassociation for 180 s. The *CsCCD4b* probe linked sensors that were soaked with PBS (no protein) were used as negative controls. The data were fitted to a 1:1 binding model and analyzed using the OCTET ANALYSIS software.

### Statistical analyses

Three replicates were performed for the experiments in this study. Results are shown as mean values ± Se. The Student's *t* test was used to determine whether the difference between two groups of data is statistically significant (*P < .05, **P < .01). GraphPad 8.0 and Microsoft Excel 2019 were used for data analyses. The summary of these analyses is shown in Supplemental Data Set 2.

### Accession numbers

Sequence data from this study can be found at Phytozome (https://phytozome-next.jgi.doe.gov/), Plaza (https://bioinformatics.psb.ugent.be/plaza/), and NCBI GenBank (https://www.ncbi.nlm.nih.gov/) under accession numbers *CsMYC2* (Ciclev10019730m), *CsMYC1* (Ciclev10019749m), CsMYC3 (Ciclev10011214m), *CsJAR1* (XM_006491175.3), *CsMPK6* (Ciclev10020481m), *MKK4* (AT1G51660.1), *CsPSY* (XM_006481880.3), *CsLCYb* (FJ516403), *CsBCH* (XM_006476445.3), *CsCCD4b* (XM_006487759.3), and *ACTIN* (XM_006486038). The accession numbers for the PUBs, BPMs, and MPKs are listed in Supplemental Figure 8.

## Supplemental data

The following materials are available in the online version of this article.

**Supplemental Figure S1**. Jasmonate production, JA-Ile production, β-citraurin accumulation, and *CsCCD4b* expression during orange fruit development and maturation.

**Supplemental Figure S2**. Exogenous MeJA promotes the color development of on-tree fruit.

**Supplemental Figure S3**. MeJA treatment increases carotenoid accumulation in orange fruit.

**Supplemental Figure S4**. MeJA treatment promotes citrus fruit coloration independently of ethylene and ABA.

**Supplemental Figure S5**. Analysis of *CsMYC2* expression.

**Supplemental Figure S6**. Localization of CsMYC2 in the nucleus.

**Supplemental Figure S7**. CsMYC2 binds the promoters of β-citraurin biosynthetic genes and CsMPK6.

**Supplemental Figure S8**. CsMPK6 attenuates the transactivation activity of CsMYC2 in citrus calli.

**Supplemental Figure S9**. Relative expression of CsMPKs, CsBPMs and CsPUBs.

**Supplemental Figure S10**. Interactions between CsMYC2 and CsMPKs, CsBPMs, and CsPUBs.

**Supplemental Figure S11**. Both the N- and C-termini of CsMYC2 interact with CsMPK6.

**Supplemental Figure S12**. CsMPK6 interacts with MKK4.

**Supplemental Figure S13**. Interactions between CsMPK6 and CsMYC2 inhibit the DNA-binding activity of CsMYC2.

**Supplemental Figure S14**. CsMPK6 attenuates the transactivation activity of CsMYC2 in Nicotiana benthamiana.

**Supplemental Figure S15**. No influence of CsMPK6 on the subcellular localization of CsMYC2.

**Supplemental Figure S16**. Carotenoid biosynthesis is upregulated by CsMYC2 and downregulated by CsMPK6.

**Supplemental Data Set S1**. Primers used in this research.

**Supplemental Table S2**. Results for statistical analyses.

## Supplementary Material

koac363_Supplementary_Data
