# Supplementary material for: Jasmonate activates a CsMPK6-CsMYC2 module that regulates the expression of β-citraurin biosynthetic genes and fruit coloration in orange (Citrus sinensis)
Source: Plant Cell. 2022 Dec 19;35(4):1167–85. doi: 10.1093/plcell/koac363 (PMC10052374; doi:10.1093/plcell/koac363)
Supplement: koac363_Supplementary_Data [file koac363_supplementary_data.zip › TPC2022RA00223R4_supplemental figures.pdf]

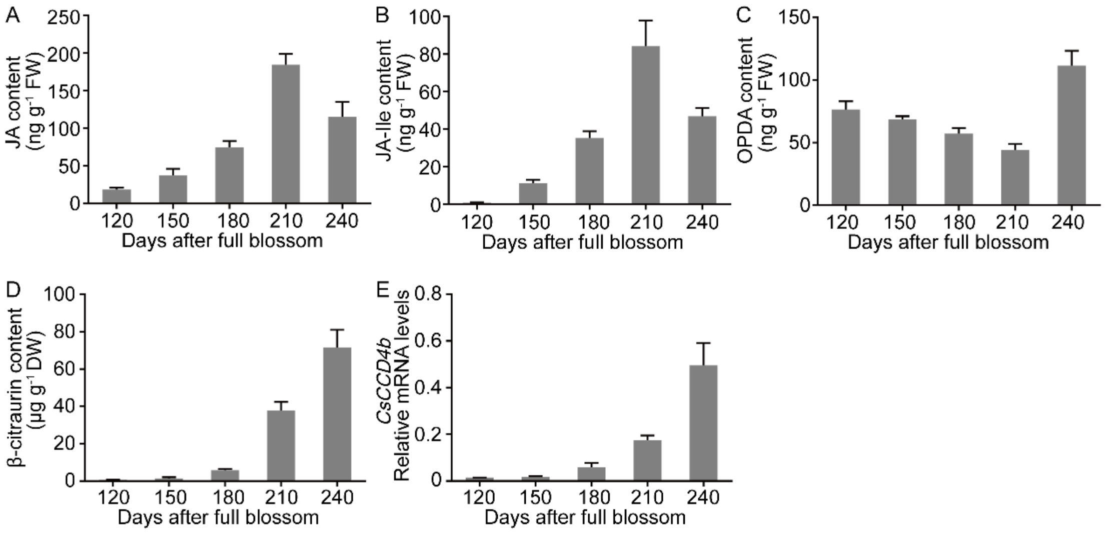

Supplemental Figure S1. Jasmonate production, JA-Ile production, OPDA production, β-citraurin accumulation, and *CsCCD4b* expression during orange fruit development and maturation. (Supports Figure 1)

(A) to (E) Fruit was collected every 30 d from 120 DAFB (days after full blossom) until 240 DAFB. Endogenous JA content (A), JA-Ile (B), 12-oxo-phytodienoic acid (OPDA) (C) and β-citraurin concentration (D) were measured, and *CsCCD4b* mRNA levels (E) were detected using RT-qPCR. The numbers under the X axes in (A) to (E) indicate the number of d after full blossom (DAFB). Peel samples from each sampling time were divided into three sets. Each set contained peels from three fruits. An independent RNA, carotenoid or plant hormone extraction from each set of peels was used as one biological replicate. Three replicates were performed. Values represent means ± SE. DW, dry weight. FW, fresh weight.

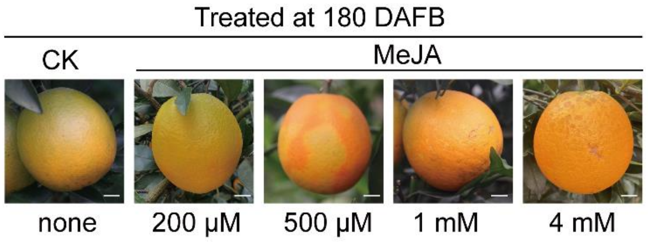

Supplemental Figure S2. Exogenous MeJA induces the color development of on-tree fruit. (Supports Figure 1)

On-tree orange fruit was sprayed with the indicated concentrations of MeJA at 180 DAFB (days after full blossom). The fruit was observed and collected at 200 DAFB. Untreated fruit was used as a control (CK, none).

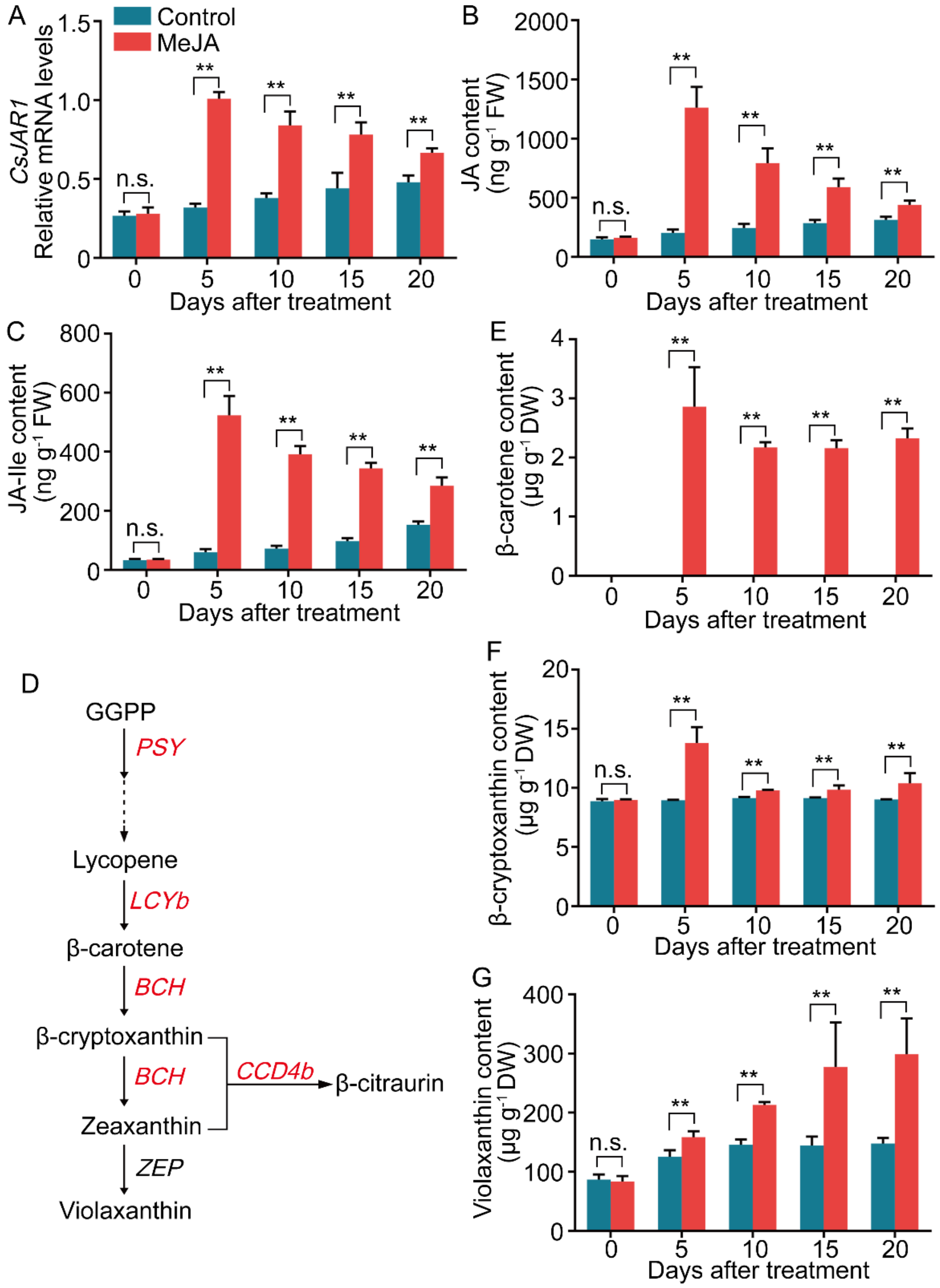

Supplemental Figure S3. MeJA treatment increases carotenoid accumulation in orange fruit. (Supports Figure 1)

(A) MeJA-induced expression of *CsJAR1.* The mRNA levels of *CsJAR1* were quantified using RT-qPCR to test whether the MeJA treatment was successful. Fruit peel samples were prepared as described in Figure 1A. Control, untreated fruit. MeJA, MeJA-treated fruit. Numbers under the X axes indicate the number of d after treatment (DAT).

(B) JA and (C) JA-Ile levels in fruit peels. Fruit peel samples were as described in Figure 1A. Control, untreated fruit. MeJA, MeJA-treated fruit. Numbers under the X axes indicate the number of d after treatment (DAT). FW, fresh weight.

(D) A simplified β-citraurin biosynthetic pathway. GGPP, geranylgeranyl diphosphate. *PSY*, phytoene synthase. *LCYb*, lycopene β-cyclase. *BCH*, β-carotene hydroxylase. *CCD4b*, carotenoid cleavage dioxygenase 4b. *ZEP*, zeaxanthin epoxidase. The dashed arrow indicates a series of reactions. The gene names in red color indicate the key genes for β-citraurin biosynthesis.

(E) to (G) β-carotene (E), β-cryptoxanthin (F), and violaxanthin (G) levels in fruit peels. Fruit peel samples were as described in Figure 1A. Control, untreated fruit. MeJA, MeJA-treated fruit. Numbers under the X axes indicate the number of d after treatment (DAT). DW, dry weight. Peel samples from each treatment were divided into three sets. Each set contained peels from three fruits. An independent plant hormone or carotenoid extraction from each set of peels was used as one biological replicate. Three replicates were performed. Values represent means ± SE. Asterisks indicate statistically significant differences, as determined by a Student's *t*-test (** P<0.01). n.s., no significant difference.

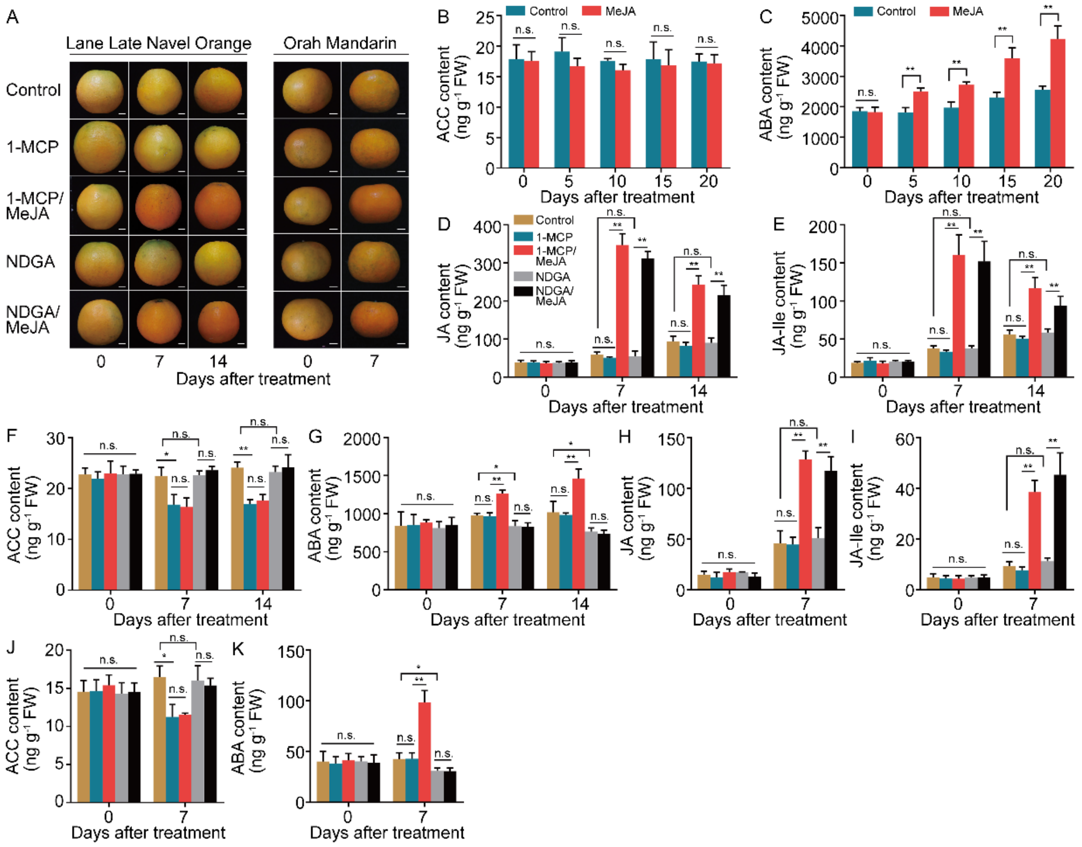

Supplemental Figure S4. MeJA treatment promotes citrus fruit coloration independently from ethylene and ABA. (Supports Figure 1)

(A) Lane Late Navel orange and Orah mandarin fruits (two β-citraurin accumulating citrus varieties) were treated with either 1-methycyclopropene (1-MCP), an ethylene inhibitor, or nordihydroguaiaretic acid (NDGA), an ABA inhibitor, to disrupt either ethylene signaling or ABA biosynthesis. The 1-MCP-treated fruit and the NDGA-treated fruit were subsequently treated with MeJA. Untreated fruits were used as controls. Late Lane Navel orange fruits were stored at room temperature for 14 d and photographed every 7 d. Orah mandarin fruits were stored at room temperature for 7 d and photographed at 7 d.

(B) ACC and (C) ABA levels in ‘Newhall’ orange fruit peels. Fruit peel samples were as described in Figure 1A. Control, untreated fruit. MeJA, MeJA-treated fruit. Numbers under the X axes indicate the number of d after treatment (DAT). FW, fresh weight.

(D) to (G) JA (D), JA-Ile (E), ACC (F) and ABA (G) levels in Lane Late Navel orange fruit peels. Fruit peel samples were as described in Supplemental

Figure 4A. Control, untreated fruit. 1-MCP, 1-MCP-treated fruit. 1-MCP/MeJA, fruit was treated firstly with 1-MCP and subsequently by MeJA. NDGA, NDGA-treated fruit. NDGA/MeJA, fruit was treated firstly with NDGA and subsequently by MeJA. Numbers under the X axes indicate the number of d after treatment (DAT). FW, fresh weight.

(H) to (K) JA (H), JA-Ile (I), ACC (J) and ABA (K) levels in Orah mandarin fruit peels. Fruit peel samples were as described in Supplemental Figure 4A. Control, untreated fruit. 1-MCP, 1-MCP-treated fruit. 1-MCP/MeJA, fruit was treated firstly with 1-MCP and subsequently by MeJA. NDGA, NDGA-treated fruit. NDGA/MeJA, fruit was treated firstly with NDGA and subsequently by MeJA. Numbers under the X axes indicate the number of d after treatment (DAT). FW, fresh weight. Peel samples from each treatment were divided into three sets. Each set contained peels from three fruits. An independent plant hormone extraction from each set of peels was used as one biological replicate. Three replicates were performed. Values represent means ± SE. Asterisks indicate statistically significant differences, as determined by a Student's *t*-test (* $P<0.05$, ** $P<0.01$). n.s., no significant difference.

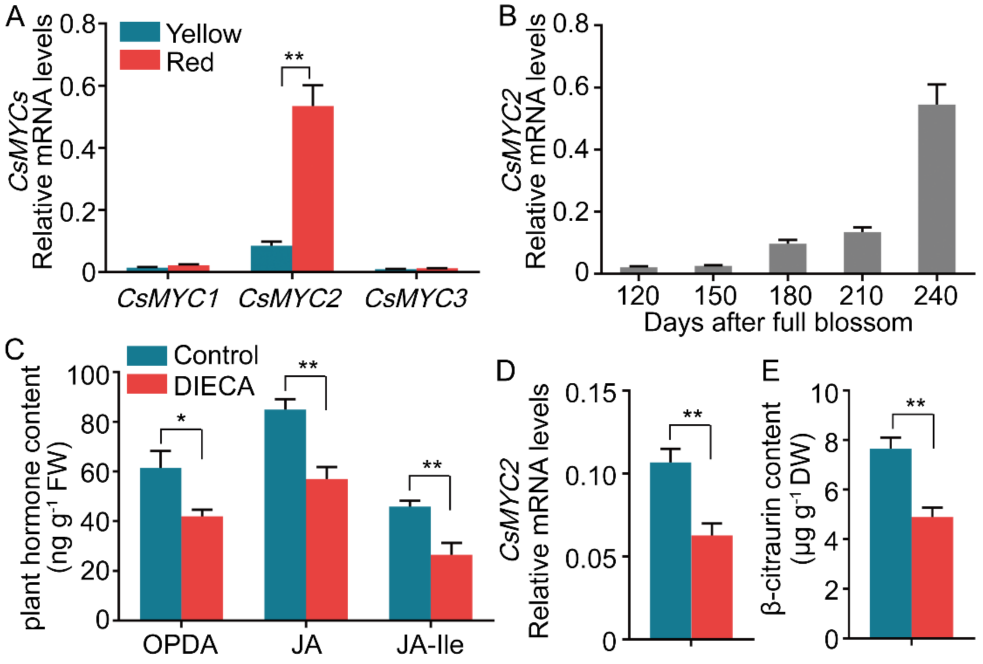

Supplemental Figure S5. Analysis of *CsMYC2* expression. (Supports Figure 2)

(A) *CsMYC* gene expression in 180 (yellow color) and 240 (red color) DAFB harvested fruit peels. Relative mRNA accumulation was quantified using RT-qPCR. Control, untreated fruit. MeJA, MeJA-treated fruit. Peel samples from each sampling time were divided into three sets. Each set contained peels from three fruits. An independent RNA extraction from each set of peels was used as one biological replicate. Three replicates were performed. Values represent means ± SE. Asterisks indicate statistically significant differences, as determined by a Student's *t*-test (** *P*<0.01).

(B) Levels of *CsMYC2* mRNA accumulation during citrus fruit development and maturation. Fruit peel samples were the same as described in Supplemental Figure 1. Peel samples from each sampling time were divided into three sets. Each set contained peels from three fruits. An independent RNA extraction from each set of peels was used as one biological replicate. Three replicates were performed.

(C) and (D) On-tree 'Newhall' orange fruits were treated with the JA inhibitor sodium diethyldithiocarbamate (DIECA) to disrupt JA biosynthesis at 160 DAFB and treated fruit was collected at 180 DAFB. Untreated fruits were used as control. Levels of OPDA, JA and JA-Ile (C), *CsMYC2* expression (D) and β-citraurin (E) were measured. Peel samples from each treatment were divided

into three sets. Each set contained peels from three fruits. An independent RNA, plant hormone or carotenoid extraction from each set of peels was used as one biological replicate. Three replicates were performed. Values represent means ± SE. Asterisks indicate statistically significant differences, as determined by a Student's *t*-test (* P<0.05, ** P<0.01).

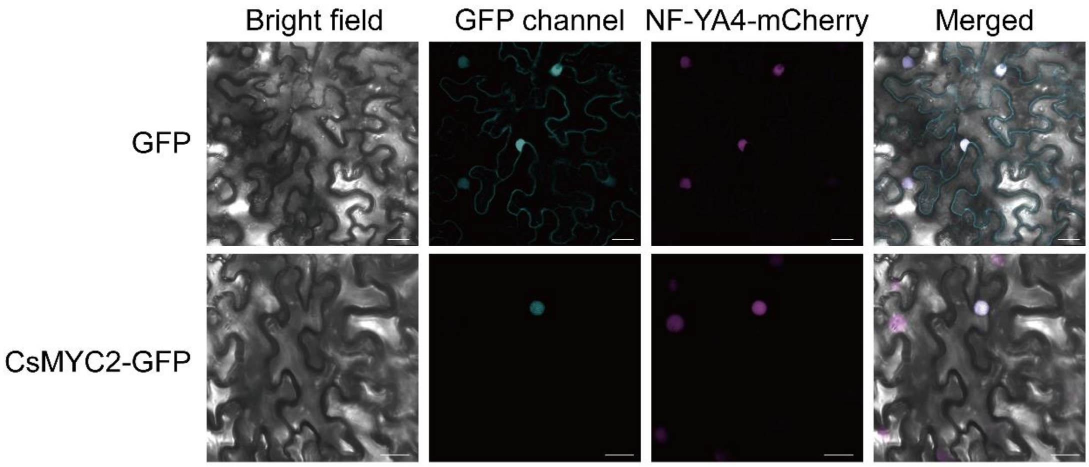

Supplemental Figure S6. Localization of CsMYC2 in the nucleus. (Supports Figure 2)

The coding sequence from *CsMYC2* was ligated downstream of the coding sequence of a GFP tag and transiently expressed using the *35S* promoter in pRI101 in *N. benthamiana* leaves (CsMYC2-GFP). *NF-YA4-mCherry* was used as a nuclear marker and co-overexpressed with CsMYC2-GFP in *N. benthamiana* leaves. Overexpression of GFP alone (GFP) was used as a control. Scale bars, 25 μm.

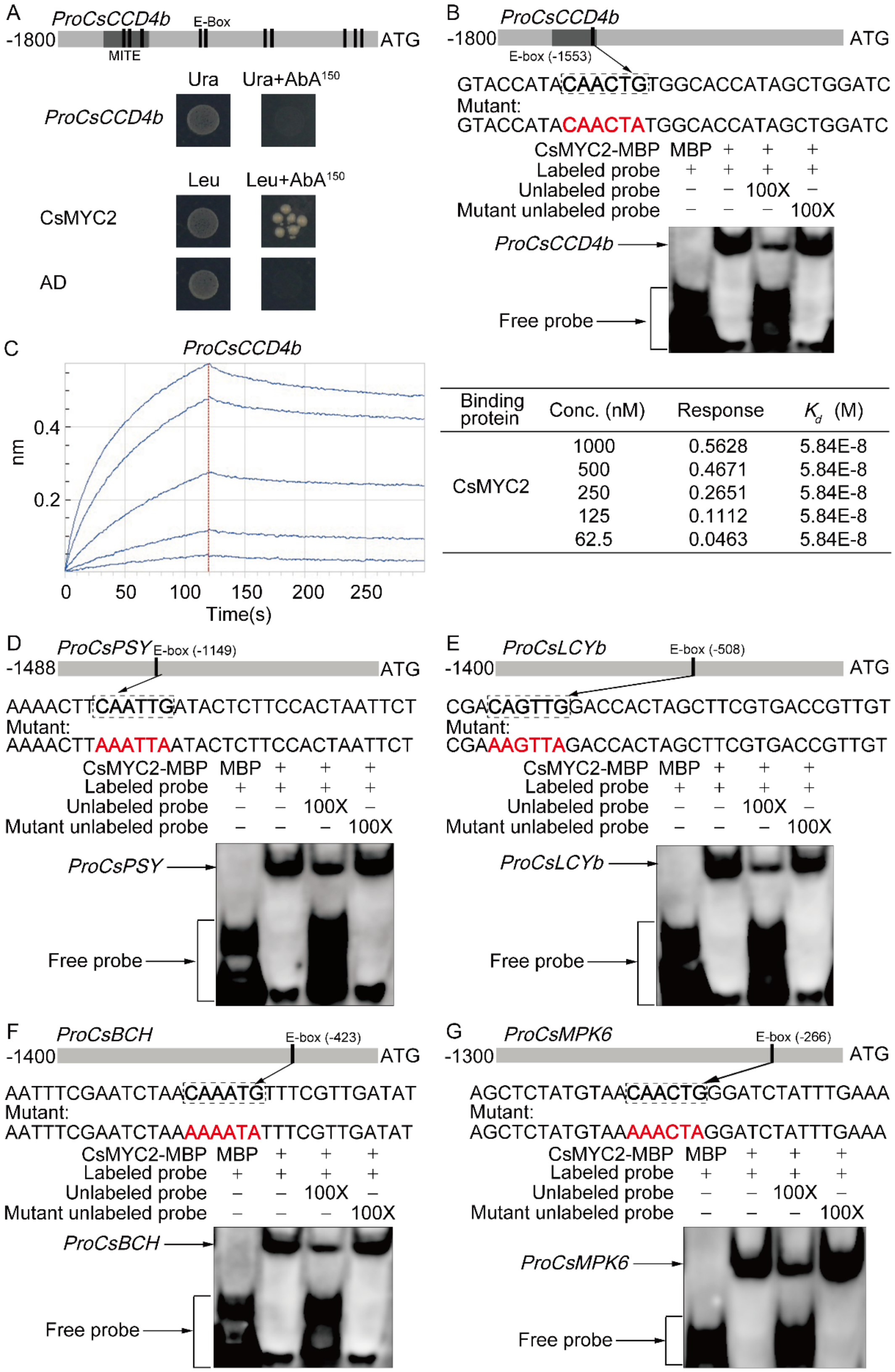

Supplemental Figure S7. CsMYC2 binds the promoters of β-citraurin biosynthetic genes and *CsMPK6*. (Supports Figure 2)

(A) CsMYC2 binds the *CsCCD4b* promoter. Yeast one-hybrid assays were

conducted by co-transforming yeast cells with plasmids that express *CsMYC2* and that contain the *CsCCD4b* promoter. AbA (Aureobasidin A), a yeast growth inhibitor, was used as a screening marker. The basal concentration of AbA was 150 ng/ml. The empty pGADT7 vector and a vector containing the *CsCCD4b* promoter were used as negative controls. E-box, bHLH binding site.

(B) CsMYC2 binds the *CsCCD4b* promoter. EMSAs were conducted with a biotin-labeled *CsCCD4b* promoter fragment containing an E-box. An unlabeled version of the same *CsCCD4b* promoter fragment was used as an unlabeled competitor at a 100-fold greater concentration than the labeled probe. For the mutant probe, the E-box was mutated to CAACTA and used as an unlabeled competitor at a 100-fold greater concentration than the labeled probe. The MBP-tagged CsMYC2 protein (4 μg) was purified. Purified MBP (4 μg) was used as a negative control.

(C) Quantification of the affinity of CsMYC2 for the *CsCCD4b* promoter. The CDS from CsMYC2 were ligated into pET30A protein expression vector, and the CsMYC2-His protein was purified. The EMSAs and the biolayer interferometry assays used the same biotin-labeled *CsCCD4b* promoter probe. For the biolayer interferometry assays, the SA biosensors were reacted with the biotin-labeled *CsCCD4b* promoter probe and then were soaked with different concentrations of the CsMYC2-His protein that ranged from 62.5 nM to 1000 nM. Sensors that were soaked with PBS were used as controls.

(D) CsMYC2 binds the *CsPSY* promoter. EMSAs were conducted with a biotin-labeled *CsPSY* promoter fragment containing an E-box. An unlabeled version of the same *CsPSY* promoter fragment was used as an unlabeled competitor at a100-fold greater concentration than the labeled probe. In the mutant probe, the E-box was mutated to AAATTA and used as an unlabeled competitor at a 100-fold greater concentration than the labeled probe. MBP-tagged CsMYC2 protein (4 μg) was purified. Purified MBP (4 μg) was used as a negative control.

(E) CsMYC2 binds the *CsLCYb* promoter. EMSAs were conducted with a biotin-labeled *CsLCYb* promoter fragment containing an E-box. An unlabeled version of the same *CsLCYb* promoter fragment was used as an unlabeled competitor probe at a 100-fold greater concentration than the labeled probe. For the mutant probe, the E-box was mutated to AAGTTA and used as an unlabeled competitor at a 100-fold greater concentration than the labeled probe. MBP-tagged

CsMYC2 protein (4 μg) was purified. Purified MBP (4 μg) was used as a negative control.

(F) CsMYC2 binds the *CsBCH* promoter. EMSAs were conducted using a biotin-labeled *CsBCH* promoter fragment containing an E-box. An unlabeled version of the same *CsBCH* promoter fragment was used as an unlabeled competitor at a 100-fold greater concentration than the labeled probe. For the mutant probe, the E-box was mutated to AAAATA and used as an unlabeled competitor at a 100-fold greater concentration than the labeled probe. MBP-tagged CsMYC2 protein (4 μg) was purified. Purified MBP (4 μg) was used as a negative control.

(G) CsMYC2 binds the *CsMPK6* promoter. EMSAs were conducted using a biotin-labeled *CsMPK6* promoter fragment containing an E-box. An unlabeled version of the same *CsMPK6* promoter fragment was used as an unlabeled competitor at a 100-fold greater concentration than the labeled probe. In the mutant probe, the E-box was mutated to AAACTA and used as an unlabeled competitor at a 100-fold greater concentration than the labeled probe. MBP-tagged CsMYC2 protein (4 μg) was purified. Purified MBP (4 μg) was used as a negative control.

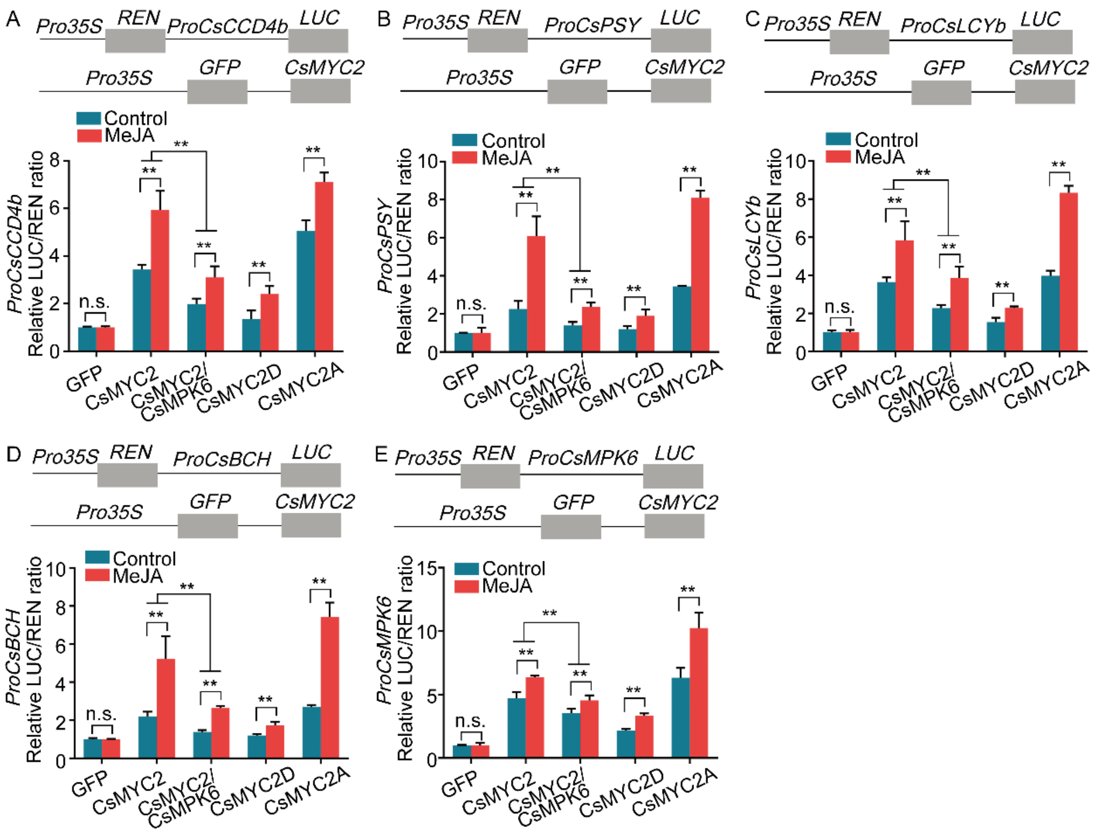

Supplemental Figure S8. CsMPK6 attenuates the transactivation activity of CsMYC2 in citrus calli. (Supports Figures 2, 3 and 5)

(A) CsMPK6 inhibits the transactivation activity of CsMYC2 on the *CsCCD4b* promoter. Plasmids encoding CsMYC2-GFP, CsMYC2-GFP with CsMPK6-myc, CsMYC2D-GFP (i.e., the phosphomimetic form) and CsMYC2A-GFP (i.e., the non-phosphorylatable form) were co-infiltrated with a plasmid containing the *CsCCD4b* promoter fused to the *LUC* reporter gene into 'Newhall' citrus calli before quantifying LUC activity. Three independent transient expression experiments were analyzed. Values represent means ± SE. Asterisks indicate statistically significant differences, as determined by a Student's *t*-test (** P<0.01). n.s., no significant difference.

(B) CsMPK6 inhibits the transactivation activity of CsMYC2 on the *CsPSY* promoter. Plasmids encoding CsMYC2-GFP, CsMYC2-GFP with CsMPK6-myc, CsMYC2D-GFP and CsMYC2A-GFP were co-infiltrated with a plasmid containing the *CsCCD4b* promoter fused to the *LUC* reporter gene into 'Newhall' citrus calli before quantifying LUC activity. Three independent transient expression experiments were analyzed. Values represent means ± SE. Asterisks indicate statistically significant differences, as determined by a

Student's *t*-test (** *P*<0.01). n.s., no significant difference.

(C) CsMPK6 inhibits the transactivation activity of CsMYC2 on the *CsLCYb* promoter. Plasmids encoding CsMYC2-GFP, CsMYC2-GFP with CsMPK6-myc, CsMYC2D-GFP and CsMYC2A-GFP were co-infiltrated with a plasmid containing the *CsLCYb* promoter fused to the *LUC* reporter gene into 'Newhall' citrus calli before quantifying LUC activity. Three independent transient expression experiments were analyzed. Values represent means ± SE. Asterisks indicate statistically significant differences, as determined by a Student's *t*-test (** *P*<0.01). n.s., no significant difference.

(D) CsMPK6 inhibits the transactivation activity of CsMYC2 on the *CsBCH* promoter. Plasmids encoding CsMYC2-GFP, CsMYC2-GFP with CsMPK6-myc, CsMYC2D-GFP and CsMYC2A-GFP were co-infiltrated with a plasmid containing the *CsBCH* promoter fused to the LUC reporter gene into 'Newhall' citrus calli before quantifying LUC activity. Three independent transient expression experiments were analyzed. Values represent means ± SE. Asterisks indicate statistically significant differences, as determined by a Student's *t*-test (** *P*<0.01). n.s., no significant difference.

(E) CsMPK6 inhibits the transactivation activity of CsMYC2 on the *CsMPK6* promoter. Plasmids encoding CsMYC2-GFP, CsMYC2-GFP with CsMPK6-myc, CsMYC2D-GFP and CsMYC2A-GFP were co-infiltrated with a plasmid containing the *CsMPK6* promoter fused to the *LUC* reporter gene into 'Newhall' citrus calli before quantifying LUC activity. Three independent transient expression experiments were analyzed. Values represent means ± SE. Asterisks indicate statistically significant differences, as determined by a Student's *t*-test (** *P*<0.01). n.s., no significant difference.

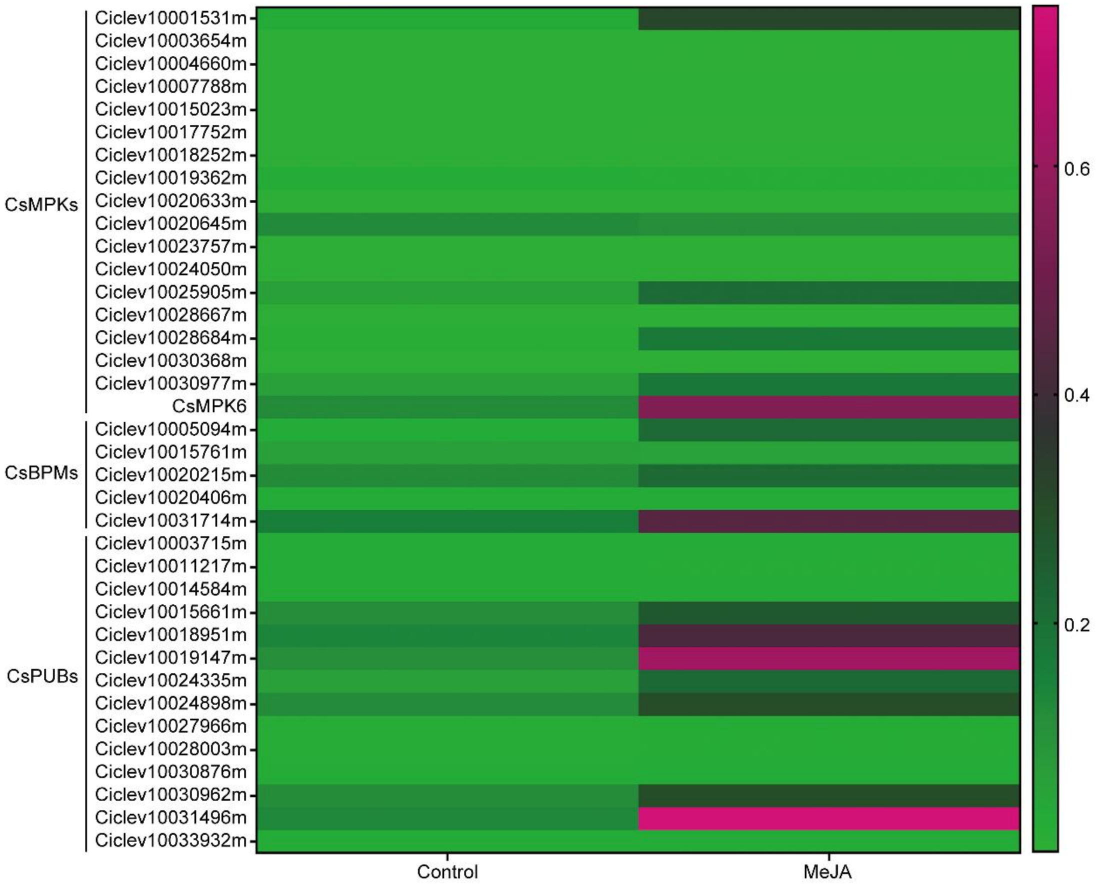

Supplemental Figure S9. Relative expression of *CsMPK*s, *CsBPM*s and *CsPUB*s. (Supports Figure 3)

The relative mRNA levels of *CsMPK*s, *CsBPM*s and *CsPUB*s were quantified using RT-qPCR in samples collected 5 DAT (days after treatment) as described in Figure 1A. Control, untreated fruit. MeJA, MeJA-treated fruit. Peel samples from each treatment were divided into three sets. Each set contained peels from three fruits. An independent RNA extraction from each set of peels was used as one biological replicate. Three replicates were performed. Genes are represented by accession numbers (except for *CsMPK6*), as shown on the left. The column on the right indicates relative gene expression levels.

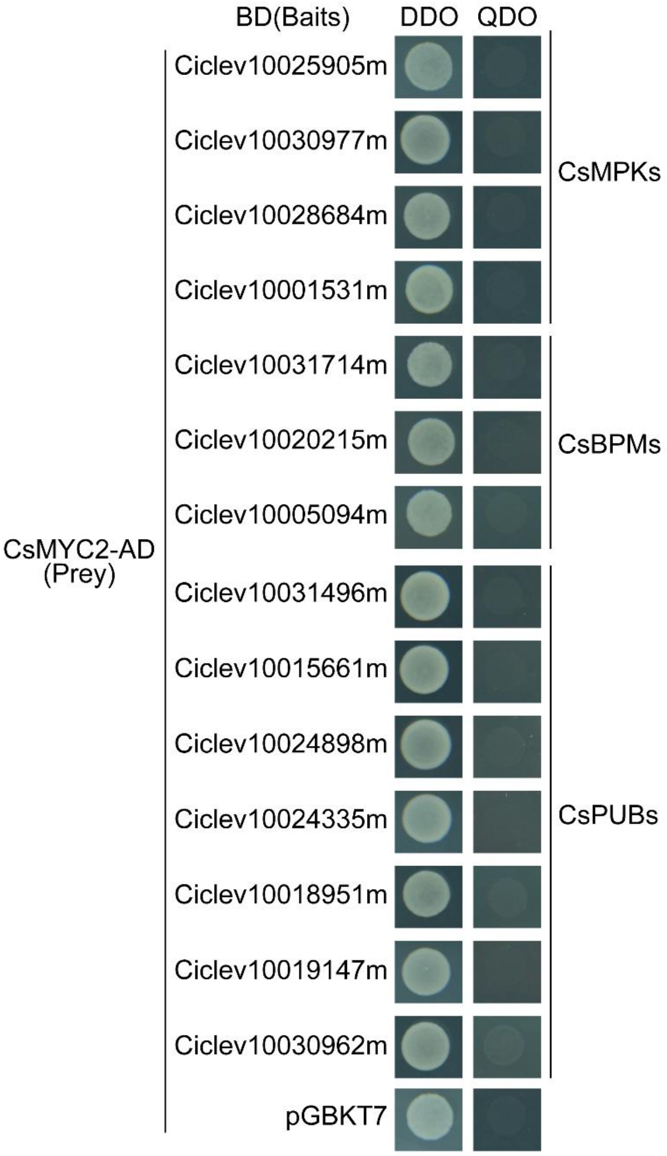

Supplemental Figure S10. Interactions between CsMYC2 and CsMPKs, CsBPMs, and CsPUBs. (Supports Figure 3)

Interactions between CsMYC2 and CsMPKs, CsBPMs, and CsPUBs were analyzed using a yeast two-hybrid (Y2H) assay. The coding sequence (CDS) from *CsMYC2* was ligated into the pGADT7 vector. The CDSs from *CsMPK*s (with the exception of *CsMPK6*), *CsBPM*s, and *CsPUB*s were separately ligated into the pGBKT7 vector. These vectors were co-transformed with the vector expressing *CsMYC2* in yeast cells. DDO, SD basal medium lacking Trp and Leu. QDO, SD basal medium lacking Trp, Leu, Ade, His. The pGBKT7 empty vector was used as a negative control. Genes are represented by accession numbers (*CsMPK6* was not included). The lack of yeast cell growth on QDO medium indicates that none of these proteins interacted with CsMYC2.

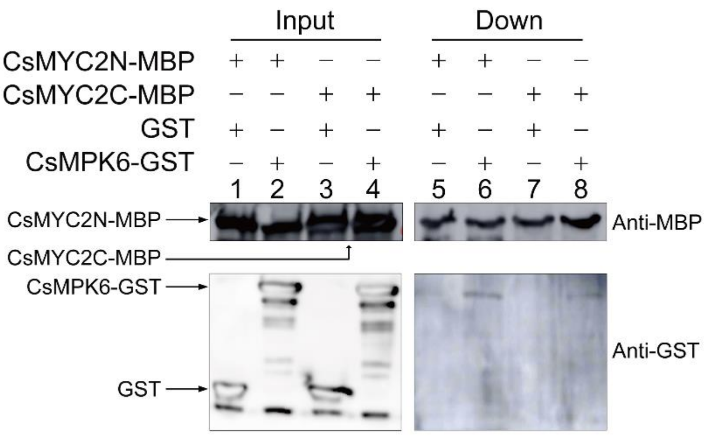

Supplemental Figure S11. Both the N- and C-termini of CsMYC2 interact with CsMPK6. (Supports Figure 3)

The interaction between CsMPK6 and the N- and C-terminus of CsMYC2 was analyzed using a pull-down assay. The N-terminus of CsMYC2 (amino acid residues 1-250, lanes 1 and 2) was fused to MBP to yield CsMYC2N-MBP. The C-terminus (amino acid residues 251-519, lanes 3 and 4) was fused to MBP to yield CsMYC2C-MBP. These fusion proteins were expressed from pMAL-C2X. The CsMYC2N-MBP and CsMYC2C-MBP fusion proteins were produced and incubated with GST or CsMPK6-GST. CsMYC2N-MBP and CsMYC2C-MBP were bound to MBP magnetic beads. Anti-GST and anti-MBP antibodies were used for the immunoblotting analysis. The band detected by the anti-GST antibody in the pulled down samples indicates that both CsMYC2N and CsMYC2C interact with CsMPK6 (lanes 6 and 8).

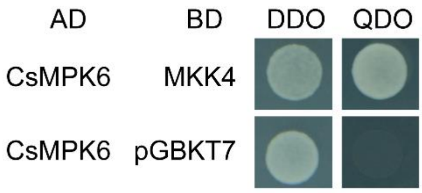

Supplemental Figure S12. CsMPK6 interacts with MKK4. (Supports Figure 4)

Interaction between CsMPK6 and MKK4DD was analyzed using a yeast two-hybrid (Y2H) assay. Yeast cells were co-transformed with plasmids that express CsMPK6 and MKK4DD. The growth of yeast cells indicates an interaction between these proteins. DDO, SD basal medium lacking Trp and Leu. QDO, SD basal medium lacking Trp, Leu, Ade, His. The pGBKT7 empty vector was used as a control.

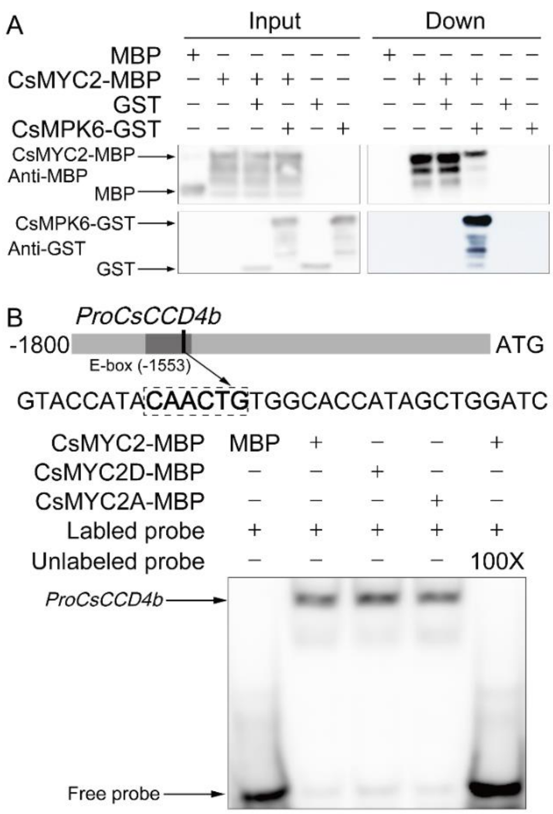

Supplemental Figure S13. Interactions between CsMPK6 and CsMYC2 inhibit the DNA binding activity of CsMYC2. (Supports Figure 5)

(A) CsMPK6 inhibits the *CsCCD4b* promoter-binding activity of CsMYC2. Biotin-labeled *CsCCD4b* promoter probes were used to pull down the CsMYC2-MBP fusion protein treated with or without CsMPK6-GST, or to pull down the MBP, GST or CsMPK6-GST protein. Anti-MBP and anti-GST antibodies were used for immunoblotting analysis. MBP, GST or CsMPK6-GST that were incubated or not incubated with CsMYC2-MBP were used as controls. Incubations with CsMPK6-GST reduced the amount of CsMYC2-MBP that was pulled down by the *CsCCD4b* promoter, which indicates that CsMPK6 inhibits the DNA-binding activity of CsMYC2.

(B) Phosphorylation does not affect the *CsCCD4b* promoter-binding activity of CsMYC2. EMSAs were conducted with a biotin-labeled *CsCCD4b* promoter fragment containing an E-box. An unlabeled version of the same *CsCCD4b* promoter fragment was used as a competitor at a 100-fold greater concentration than the labeled probe. MBP-tagged CsMYC2 (2 μg), CsMYC2D (i.e., the phosphomimetic form, 2 μg), and CsMYC2A (non-phosphorylatable form, 2 μg) were purified. Purified MBP (2 μg) was used as a negative control.

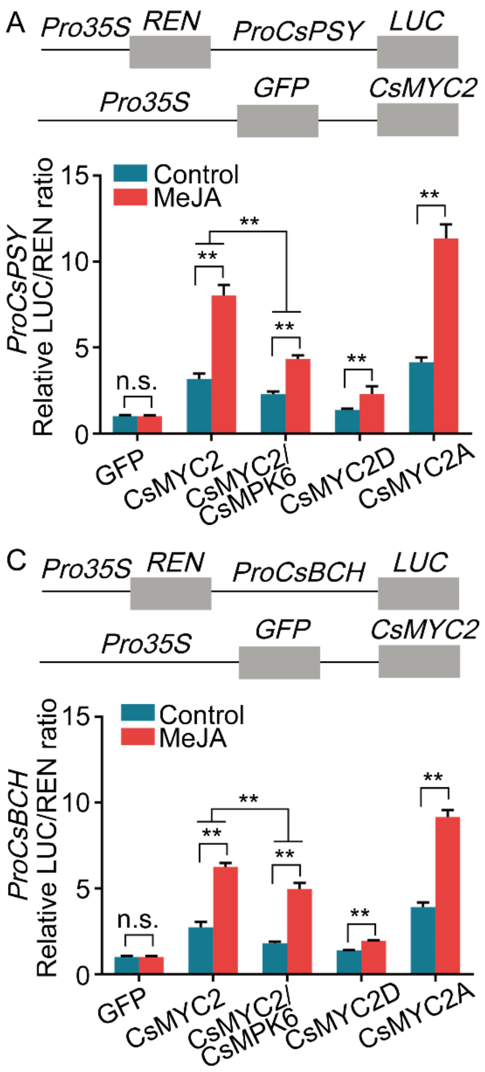

Supplemental Figure S14. CsMPK6 attenuates the transactivation activity of CsMYC2 in *Nicotiana benthamiana*. (Supports Figure 5)

(A) CsMPK6 inhibits the transactivation activity of CsMYC2 on the *CsPSY* promoter. Plasmids encoding CsMYC2-GFP, CsMYC2-GFP withCsMPK6-myc, CsMYC2D-GFP (i.e., the phosphomimetic form) and CsMYC2A-GFP (i.e., the non-phosphorylatable form) were co-infiltrated with a plasmid containing the *CsCCD4b* promoter fused to the *LUC* reporter gene into *N. benthamiana* leaves before quantifying LUC activity. Three independent transient expression experiments were analyzed. Values represent means ± SE. Asterisks indicate statistically significant differences, as determined by a Student's *t*-test (** *P*<0.01). n.s., no significant difference.

(B) CsMPK6 inhibits the transactivation activity of CsMYC2 on the *CsLCYb* promoter. Plasmids encoding CsMYC2-GFP, CsMYC2-GFP with CsMPK6-myc,

CsMYC2D-GFP and CsMYC2A-GFP were co-infiltrated with a plasmid containing the *CsLCYb* promoter fused to the *LUC* reporter gene into *N. benthamiana* leaves before quantifying LUC activity. Three independent transient expression experiments were analyzed. Values represent means ± SE. Asterisks indicate statistically significant differences, as determined by a Student's *t*-test (** P<0.01). n.s., no significant difference.

(C) CsMPK6 inhibits the transactivation activity of CsMYC2 on the *CsBCH* promoter. Plasmids encoding CsMYC2-GFP, CsMYC2-GFP with CsMPK6-myc, CsMYC2D-GFP and CsMYC2A-GFP were co-infiltrated with a plasmid containing the *CsBCH* promoter fused to the LUC reporter gene into *N. benthamiana* leaves before quantifying LUC activity. Three independent transient expression experiments were analyzed. Values represent means ± SE. Asterisks indicate statistically significant differences, as determined by a Student's *t*-test (** P<0.01). n.s., no significant difference.

(D) CsMPK6 inhibits the transactivation activity of CsMYC2 on the *CsMPK6* promoter. Plasmids encoding CsMYC2-GFP, CsMYC2-GFP with CsMPK6-myc, CsMYC2D-GFP and CsMYC2A-GFP were co-infiltrated with a plasmid containing the *CsMPK6* promoter fused to the *LUC* reporter gene into *N. benthamiana* leaves before quantifying LUC activity. Three independent transient expression experiments were analyzed. Values represent means ± SE. Asterisks indicate statistically significant differences, as determined by a Student's *t*-test (** P<0.01). n.s., no significant difference.

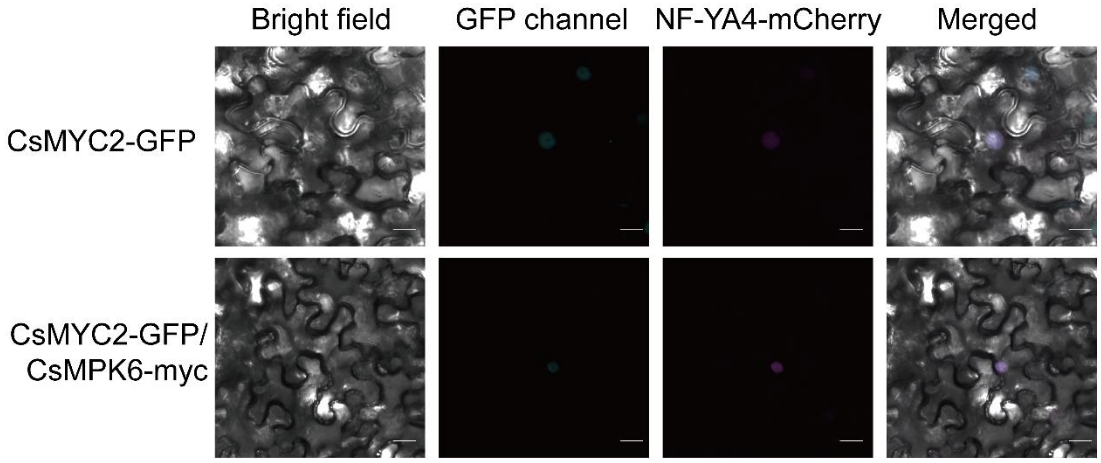

Supplemental Figure S15. No influence of CsMPK6 on the subcellular localization of CsMYC2. (Supports Figure 5)

The coding sequence of *CsMYC*2 was ligated downstream of the coding sequence for a GFP tag and expressed under the control of the *35S* promoter in pRI101.The coding sequence of *CsMPK6* was ligated downstream of the coding sequence of a myc tag and expressed under the control of the *35S* promoter in pCAMBIA1305. CsMYC2-GFP and CsMYC2-myc were transiently expressed in *N. benthamiana* leaves (CsMYC2-GFP/CsMPK6-myc). *NF-YA4-mCherry* was used as a nuclear marker and co-overexpressed with CsMYC2-GFP/CsMPK6-myc in *N. benthamiana* leaves. Expression of only CsMYC2-GFP was used as a control. Scale bars, 25 μm.

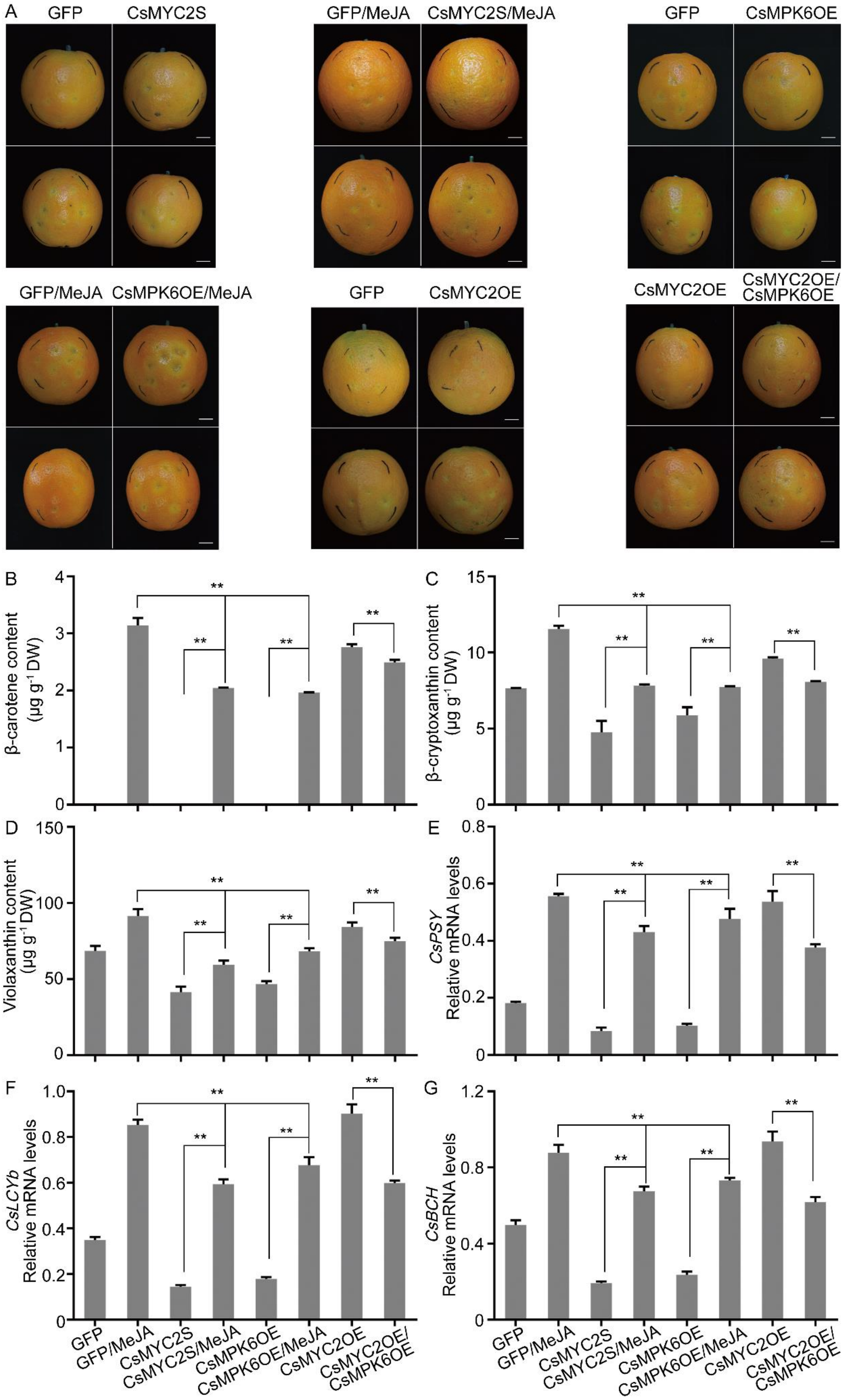
A
GFP
CsMYC2S
GFP/MeJA
CsMYC2S/MeJA
GFP
CsMPK6OE
GFP/MeJA
CsMPK6OE/MeJA
GFP
CsMYC2OE
CsMYC2OE
CsMYC2OE/
CsMPK6OE
B
β-carotene content
(μg g-1 DW)
C
β-cryptoxanthin content
(μg g-1 DW)
D
Violaxanthin content
(μg g-1 DW)
E
CsPSY
Relative mRNA levels
F
CsLCYb
Relative mRNA levels
G
CsBCH
Relative mRNA levels
**
GFP
GFP/MeJA
CsMYC2S
CsMYC2S/MeJA
CsMPK6OE
CsMPK6OE/MeJA
CsMYC2OE
CsMYC2OE/
CsMPK6OE

Supplemental Figure S16. Carotenoid biosynthesis is upregulated by CsMYC2 and downregulated by CsMPK6. (Supports Figure 6)
(A) to (G) Silencing or overexpressing *CsMYC2* (CsMYC2S, CsMYC2OE) and overexpressing *CsMPK6* (CsMPK6OE) in on-tree citrus fruit at 195 DAFB in 2021 using *Agrobacterium*-mediated transient transformation (A, phenotypes). MeJA treatment was performed 3 d after infiltration, and fruit was harvested 15 d after infiltration. Fruit expressing GFP was used as a control. Scale bars, 1 cm. β-carotene (B), β-cryptoxanthin (C) and violaxanthin (D) contents were measured, and *CsPSY* (E), *CsLCYb* (F) and *CsBCH* (G) mRNA levels in infiltrated fruit were measured using RT-qPCR. DW, dry weight. Peel samples from each group of infiltrated fruits were divided into three sets. Each set contained peels from five fruits. An independent RNA or carotenoid extraction from each set of peels was used as one biological replicate. Three replicates were performed. Values represent means ± SE. Asterisks indicate statistically significant differences, as determined by a Student's *t*-test (** $P<0.01$). n.s., no significant difference.
